# Sortilin‐Mediated Inhibition of TREK1/2 Channels in Primary Sensory Neurons Promotes Prediabetic Neuropathic Pain

**DOI:** 10.1002/advs.202310295

**Published:** 2024-04-16

**Authors:** Wei Sun, Fan Yang, Yan Wang, Yan Yang, Rui Du, Xiao‐Liang Wang, Zhi‐Xin Luo, Jun‐Jie Wu, Jun Chen

**Affiliations:** ^1^ Institute for Biomedical Sciences of Pain Tangdu Hospital The Fourth Military Medical University Xi'an Shaanxi Province 710038 P. R. China; ^2^ State Key Laboratory of Oral & Maxillofacial Reconstruction and Regeneration National Clinical Research Center for Oral Diseases Shaanxi Key Laboratory of Stomatology Department of Orthodontics, School of Stomatology The Fourth Military Medical University Xi'an Shaanxi Province 710032 P. R. China; ^3^ Present address: Sanhang Institute for Brain Science and Technology (SiBST) School of Medical Research, Northwestern Polytechnical University (NPU) Xi'an Shaanxi 710129 P. R. China

**Keywords:** dorsal root ganglion, hyperexcitability, prediabetic neuropathic pain, Sortilin, TREK1, TREK2

## Abstract

Neuropathic pain can occur during the prediabetic stage, even in the absence of hyperglycemia. The presence of prediabetic neuropathic pain (PDNP) poses challenges to the management of individuals with prediabetes. However, the mechanisms underlying this pain remain unclear. This study aims to investigate the underlying mechanism and identify potential therapeutic targets of PDNP. A prediabetic animal model induced by a high‐energy diet exhibits both mechanical allodynia and thermal hyperalgesia. Furthermore, hyperexcitability and decreased potassium currents are observed in the dorsal root ganglion (DRG) neurons of these rats. TREK1 and TREK2 channels, which belong to the two‐pore‐domain K^+^ channel (*K*_2P_) family and play an important role in controlling cellular excitability, are downregulated in DRG neurons. Moreover, this alteration is modulated by Sortilin, a molecular partner that modulates the expression of TREK1. The overexpression of Sortilin negatively affects the expression of TREK1 and TREK2, leading to increased neuronal excitability in the DRG and enhanced peripheral pain sensitivity in rats. Moreover, the downregulation of Sortilin or activation of TREK1 and TREK2 channels by genetic or pharmacological approaches can alleviate PDNP. Therefore, targeting the Sortilin‐mediated TREK1/2 pathway may provide a therapeutic approach for ameliorating PDNP.

## Graphical abstract

This study identifies the roles of TREK1 and TREK2 in prediabetic neuropathic pain and investigates the modulation of Sortilin within these channels. It suggests that Sortilin overexpression leads to a decrease in TREK1 and TREK2 expression, which in turn contributes to the modulation of neural hyperexcitability and nociceptive hypersensitivity in rats with prediabetes.

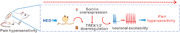

## 1 Introduction

Individuals who have impaired glucose tolerance (IGT) or insulin resistance but do not fulfill the diagnostic criteria for type 2 diabetes are usually considered as having prediabetes.^[^ ^1^, ^2^ ^]^ Recently, neuropathy has been reported to manifest in the initial phases of glucose dysmetabolism. Contrary to common belief that the neuropathic complications of diabetes develop only after long‐term hyperglycemia, prediabetic neuropathy (PDN) appears to occur prior to the onset of hyperglycemia. Additionally, several studies have highlighted the occurrence of chronic axonal polyneuropathy in the prediabetic stage, which serves as a major cause of neuropathic pain and acts as an initiating factor for diabetic foot ulceration. Therefore, many individuals with prediabetes may experience peripheral neuropathy and/or neuropathic pain.^[^ ^3^, ^4^ ^]^ This neuropathy is characterized by the distal‐to‐proximal degeneration of peripheral nerves, which causes pain, weakness, and eventual loss of feeling. Approximately, 50–60% of individuals with prediabetes suffer persistent prediabetic neuropathic pain (PDNP) and remain lacking effective therapies owing to an inadequate understanding of the underlying mechanisms involved in PDNP.^[^ ^1^, ^5^ ^]^

Prediabetic animal models induced by a high‐energy diet (HED) have been widely used to explore the pathogenesis of prediabetes and PDNP because of their reproducibility and clinical relevance. IGT and increased insulin concentrations gradually emerged in rats after HED feeding.^[^ ^6^ ^]^ Significant painful neuropathy and deficiencies in nerve conduction were also observed in these animals.^[^ ^7^ ^]^ However, over the past few decades, there has been a lack of neural electrophysiological and histological evidence to support the existence of PDNP in prediabetes.^[^ ^6^, ^8^ ^]^ In recent years, investigations have been conducted on the morphological features of peripheral nerves under prediabetic conditions. These studies indicate that the myelin sheaths are loosened, disordered, and partially broken up into vesicle‐like membrane fragments. Additionally, axonal lesions have been observed in unmyelinated nerve fibers using ultrastructural pathology.^[^ ^9^, ^10^ ^]^ Moreover, several studies investigating prediabetic neural function have reported significantly lower Ca^2+^ levels in the sensory axons, slower nerve conduction, and impaired neuronal mitochondrial function in the peripheral nervous system (PNS) of the PDNP animal model.^[^ ^11^, ^12^, ^13^ ^]^ The development of prediabetes‐associated neuropathy may also be influenced by intricate pathogenic factors. Currently, the molecular processes underlying the effects of PDNP remain unclear.

Hyperexcitability of the soma and axons of dorsal root ganglion (DRG) neurons has been proposed as a crucial parameter in determining peripheral pain sensitization. Neural excitability and membrane‐active properties can be influenced by the sequential activation of voltage‐dependent Na^+^ and K^+^ channels, which are two types of significant ion channels involved in depolarization and repolarization.^[^ ^14^, ^15^ ^]^ The two‐pore‐domain K^+^ channel (*K*_2P_) is a crucial branch of the K^+^ channel that serves as the primary molecular correlate of the background potassium current and plays an important role in controlling cellular excitability. In mammals, the *K*_2P_ family consists of 15 different members grouped into six subfamilies based on sequence similarity. Among these subfamilies, the TWIK‐related K1 channel (TREK) subfamily is widely expressed in the nervous system, particularly in the PNS, and includes three members: TREK‐1, TREK‐2, and TWIK‐related arachidonic acid‐activated K1 channel (TRAAK).^[^ ^16^, ^17^ ^]^ These members exhibit a sequence homology exceeding 78% and share some common mechanisms of activation. These channels can be activated by various stimuli, including membrane stretching and swelling, cellular acidification (pHi), elevated temperatures, and polyunsaturated fatty acids, such as arachidonic acid, lysophospholipids, and phosphatidylinositol 4, 5‐bisphosphate, as well as volatile anesthetics such as halothane and isoflurane.^[^ ^18^, ^19^, ^20^ ^]^ Therefore, these channels play a role in a variety of physiological and pathological processes, including anesthesia, neuroprotection, depression, and pain.^[^ ^21^ ^]^ In terms of cellular electrophysiological properties, these three *K*_2P_ channels significantly contribute to the background K^+^ currents in DRG neurons. The predominant generator of this background K^+^ current is found to be the TREK2 channel (69%), followed by TREK‐1 at 12%, and TRAAK at 3%.^[^ ^22^ ^]^ Recent evidence strongly suggests that a variety of TREK channels play crucial roles in the processing of chronic pain. Utilizing transgenic technology, it has been discovered that mice lacking either TREK1 or TREK2 channels exhibit notable hypersensitivity to both thermal and mechanical stimuli.^[^ ^23^, ^24^ ^]^ Furthermore, research conducted on animal models of pain has shown the crucial role of TREK1 and TREK2 channels in modulating nociceptive hypersensitivity in various conditions such as migraine, spinal nerve ligation (SNL)‐induced neuropathic pain, formalin‐induced inflammatory pain, prostatitis pain, and oxaliplatin‐induced pain. Therefore, these channels may serve as molecular targets for analgesia.^[^ ^25^, ^26^, ^27^, ^28^, ^29^ ^]^ Nevertheless, the roles of TREK1 and TREK2 channels in neuropathic pain caused by prediabetes remain largely unknown. In addition, the expression of these TREK channels can be modulated via intracellular sorting and scaffolding proteins. For example, Sortilin, microtubule‐associated protein 2, and coatomeric protein complex 1 are potential regulators of TREK1 expression.^[^ ^30^, ^31^, ^32^ ^]^ Sortilin, a sorting protein, is abundantly expressed in the central nervous system (CNS) and other cell types such as adipocytes, hepatocytes, and macrophages. It plays a crucial role in protein trafficking and the modulation of glucose and lipid metabolism.^[^ ^33^ ^]^ In the nervous system, Sortilin has been reported to regulate the expression of TREK1 on the membrane of cortical neurons and may serve as a target for molecular antidepressants.^[^ ^34^ ^]^ However, the mechanisms through which Sortilin regulates TREK1 and TREK2 expression in prediabetic DRG neurons are not fully understood.

In conclusion, manipulating the expression of TREK1/2 channels in sensory neurons may affect neuronal excitability and nociceptive sensation and this pathway should be considered a potential therapeutic target for the treatment of pain. In this study, we identified the roles of TREK1 and TREK2 in PDNP and investigated the modulation of Sortilin within these channels. Our findings suggest that Sortilin overexpression leads to a decrease in TREK1 and TREK2 expression, which in turn contributes to the modulation of neural hyperexcitability and nociceptive hypersensitivity in rats with prediabetes.

## 2 Results

### 2.1 Establishing a Prediabetic Animal Model

Prediabetes was induced in rats using a HED. Remarkable metabolic disorders and pain sensitivity were observed in these rats. Consistent with our previous reports,^[^ ^10^ ^]^ the body weights of prediabetic and control rats did not significantly change (Figure S1A, Supporting Information) up to the 65th day after a HED, although the body weight of rats increased with the feeding time course. Additionally, up to the 25th day and the 65th day on a HED, the fasting blood glucose (FBG) levels of rats were not significantly elevated (Figure S1B, Supporting Information). Compared with the control rats, the HED‐fed rats showed a significant increase in fasting plasma insulin levels over time (Figure S1C, Supporting Information), as measured on the 65th and 80th days after HED. Insulin resistance was estimated using mathematical models based on fasting insulin and glucose levels, which were determined by a homeostasis model assessment for insulin resistance (HOMA‐IR). Further analysis indicated that HOMA‐IR was significantly elevated on the 65th day (an increase of 5.39 ± 2.51) and the 80th day (an increase of 10.93 ± 4.03) after a HED as compared to that of a normal diet (Figure S1D, Supporting Information). To determine whether neuropathic pain occurred in this prediabetic phase, as per our previous findings, painful behavioral tests were performed, and the mechanical threshold in response to von Frey filaments was detected. Mechanical allodynia developed in rats fed a HED. Notably, the mechanical paw withdrawal threshold (PWMT) of the bilateral paws of the rats decreased from the 50th day following the HED (Figure S2A,B, Supporting Information).

Furthermore, thermal pain was assessed by measuring paw withdrawal response to a radiant heat source. Similarly, rats fed a HED exhibited thermal hyperalgesia. The bilateral thermal paw withdrawal latency (PWTL) of the rats was found to have reduced on the 65th day following the HED (Figure S2C,D, Supporting Information). Therefore, pain hypersensitivity may begin during the prediabetic phase, even with normal blood glucose levels and insulin resistance. Rats with normal FBG levels but elevated serum insulin levels and decreased PWMT and PWTL were used for further assays.

### 2.2 Detection of Hyperexcitability and Attenuated Total Potassium Current in DRG Neurons from Prediabetic Rats in Pain

**Figure 1:**
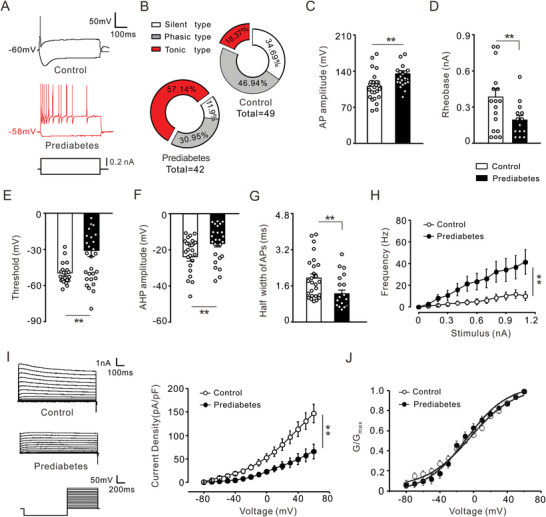
Remarkable hyperexcitability and reduced potassium current were observed in DRG cells from prediabetic rats. A) Representative traces show the spike firings in response to a depolarizing current step (50 pA step, 500 ms duration) in DRG neurons from control and prediabetic rats. The protocol for recording action potentials is depicted at the bottom. B) Circular charts display the proportions of silent type cells, tonic firing type cells, and phasic (single) firing type cells in the recording DRG neurons from the control (*n* = 49 cells) and prediabetic group (*n* = 42 cells). C) Enhanced action potentials amplitude was detected in prediabetic DRG neurons. **p < 0.01, prediabetes versus control, Mann Whitney *U* test, *n* = 26 cells per group. D) The rheobase for the first action potential firing was significantly reduced in the prediabetic group compared to the control, as shown by the histogram. **p < 0.01, prediabetes versus control, Mann Whitney *U* test, 14 cells for prediabetes versus 18 cells for control. E) The threshold of action potential was more hyperpolarized in the prediabetic group than in the control group. **p < 0.01, prediabetes versus control, Mann Whitney *U* test, *n* = 21 cells for prediabetes versus 31 cells for control. F) The amplitude of after‐hyperpolarization was measured from the resting level to the nadir. The peak amplitude of after‐hyperpolarization was lower in the prediabetic group than in the control group. **p < 0.01, prediabetes versus control, Mann Whitney *U* test, *n* = 26 cells per group. G) The half‐width of action potential was shorter in the prediabetic group. **p < 0.01, prediabetes versus control, Mann Whitney *U* test, *n* = 21 cells for prediabetes versus 26 cells for control. H) The plots showed a significant increase in firing frequency from the prediabetic group (*n* = 21 cells) compared to the control (*n* = 35 cells). **p < 0.01, Two‐way ANOVA RM. I) Left: representative traces of potassium current are elicited by 700 ms depolarizing commands ranging from −80 to 50 mV. The protocol for recording *I*_K_ is shown at the bottom. Right: *I–V* relations curves of *I*_K_ were obtained from both the control and prediabetic groups. Quantitative analysis revealed that the amplitude of *I*_K_ at 50 mV was greatly increased in DRG neurons (*n* = 17 cells) from prediabetic rats compared to controls (*n* = 26 cells). **p < 0.05, Two‐way ANOVA RM. J) The plots show the voltage dependence of activation curves of *I*_K_ from prediabetic (*n* = 17 cells, filled circles) and control (*n* = 26 cells, opened circles) neurons. To assess voltage‐dependent activation, the voltage commands were applied (ranging from −80 to 60 mV) in 10 mV increments.

The hyperexcitability of primary afferent sensory neurons is regarded as an important indicator of peripheral pain sensitization, which is defined as the increased responsiveness of sensory neurons in the PNS to normal or subthreshold stimuli. In the present study, we used intact DRGs to perform whole‐cell recordings. We then analyzed the neural membrane properties, excitability, and potassium currents in painful prediabetic and control rats. These data illustrate the increased excitability of DRG neurons in rats with prediabetes. **Figure** **1**A shows representative neural firing from the prediabetic and control groups, with action potentials elicited under a current clamp using a 500 ms command current ranging from −150 to 300 pA at 50 pA intervals. The firing patterns of the DRG neurons were analyzed in both prediabetic and control rats. In the PNS, tonic firing is defined as a sustained discharge to a stimulus, whereas phasic firing refers to a transient response with one or a few action potentials at the onset of a stimulus followed by accommodation. In this study, the proportions of different firing patterns were calculated and were shown in pie charts. The DRG neuronal recordings from the control group revealed that 18.37%, 46.94%, and 34.69% exhibited tonic, phasic, and silent firing. Interestingly, under prediabetic conditions, the percentage of tonic firing type neurons increased to 57.14%, while the percentages of phasic firing type and silent type neurons decreased to 30.95% and 11.9%, respectively (Figure 1B). The amplitude of the action potential in the prediabetic group was significantly higher than that in the control group (Figure 1C). The rheobase of prediabetic DRG neurons was remarkably lower than that of control neurons (Figure 1D). The threshold for action potential shifted more toward hyperpolarization in prediabetic DRG neurons than in control neurons (Figure 1E). In addition, prediabetic DRG neurons exhibited a smaller amplitude of after hyperpolarization potential (AHP) and a shorter half‐width of action potential than control neurons (Figure 1F,G). More importantly, the firing frequency of prediabetic DRG neurons significantly increased from 3.19 ± 2.24 to 41.57 ± 10.34 Hz in response to increasingly stronger current injections (Figure 1H). These results demonstrate noticeable hyperexcitability in DRG cells from prediabetic rats. To further investigate the ion current mechanism underlying this neuronal hyperexcitability, we examined the potassium currents in prediabetic DRG neurons, which regulate neuronal excitability by stabilizing the resting membrane potential (RMP) and modulating the action potential shape and frequency. The cells were held at −80 mV, and voltage steps were imposed from −80 to +50 mV in 10 mV increments. The total potassium current (*K*v) was obtained by using a protocol with a 1 s prepulse at −120 mV, which could completely inactivate the potassium current (Figure 1I, left panel). The average current density for the total *I*_K_ was significantly lower in the prediabetic group than in the control group (Figure 1I, right panel). The kinetics of activation of total *I*_K_ were calculated and shown in Figure 1J. Prediabetes caused a significant depolarizing shift of 2 mV in the half‐activation potential (*V*_1/2_) of the total *I*_K_ (Figure 1J).

### 2.3 Downregulated Expression of TREK1 and TREK2 in DRG Neurons of Rats with HED

**Figure 2:**
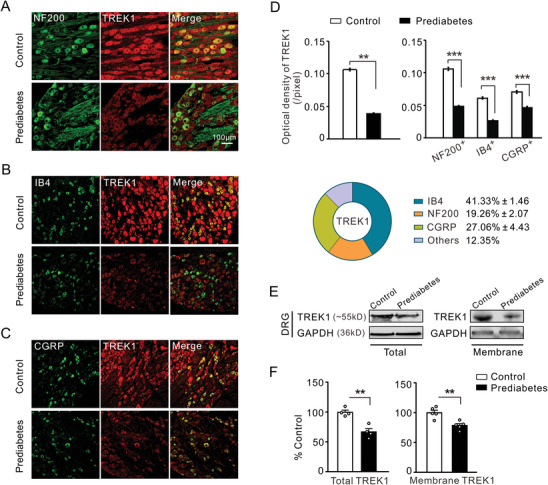
Characterization of the expression profile of TREK1 in DRG neurons reveals that the expression of TREK1 in NF200‐, IB4‐, and CGRP‐positive neurons is decreased in prediabetic rats compared to controls. A) Double immunofluorescence staining of DRG neurons with large cell marker NF200 (green) and TREK1 antibody (red) in control rats (top) and prediabetic rats (bottom), X20 magnification. B) Double immunofluorescence staining of DRG neurons with IB4 (green) and TREK1 antibody (red) in control rats (top) and prediabetic rats (bottom), X20 magnification. C) Immunofluorescence labeling of CGRP (green) and TREK1 antibody (red) in DRG neurons from control rats (top) and prediabetic rats (bottom), X20 magnification. D) Quantitative analysis showed the average immunofluorescence intensity of TREK1 significantly decreased in prediabetes groups compared to controls (left panel). The right panel shows the immunofluorescence intensity of TREK1 in NF200‐, IB4‐, and CGRP‐positive DRG neurons. The bottom panel shows the percentage of TREK1 immunoreactivity in NF200‐, IB4‐, and CGRP‐positive DRG neurons. **p < 0.01, **p < 0.001, Mann Whitney *U* test, *n* = 4 rats per group. E) The representative bands of TREK1 in DRG were detected by western blot derived from the whole cells (upper) and cellular membrane (bottom), respectively. F) Grouped data show that the expression of TREK1 in both whole cells and the cellular membrane was downregulated in the prediabetes group compared to the control group. **p < 0.01, Independent Samples *t*‐test, *n* = 4–5 rats per group. Scale bars: (A), (B), (C) = 100 µm.

**Figure 3:**
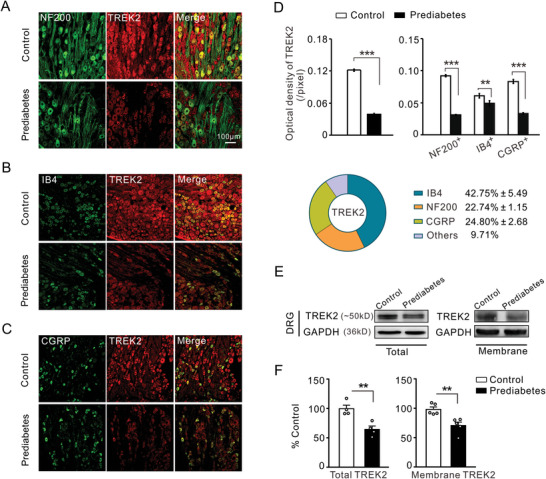
Characterization of the expression profile of TREK2 in DRG reveals that the expression of TREK2 in NF200‐, IB4‐, and CGRP‐ positive DRG neurons is decreased in prediabetic rats compared to controls. A) Double immunofluorescence staining of DRG neurons with large cell marker NF200 (green) and TREK2 antibody (red) in control rats (top) and prediabetic rats (bottom), X20 magnification. B) Double immunofluorescence staining of DRG neurons with IB4 (green) and TREK2 antibody (red) in control rats (top) and prediabetic rats (bottom), X20 magnification. C) Immunofluorescence labeling of CGRP (green) and TREK2 antibody (red) in DRG neurons from control rats (top) and prediabetic rats (bottom), X20 magnification. D) Quantitative analysis shows the average immunofluorescence intensity of TREK2 significantly decreased in the prediabetes group compared to the control group (left panel). The right panel shows the immunofluorescence intensity of TREK2 in NF200‐, IB4‐, and CGRP‐positive DRG neurons. The pie chart shows the percentage of TREK2 immunoreactivity in NF200‐, IB4‐, and CGRP‐positive DRG neurons. **p < 0.01, ***p < 0.001, Mann Whitney *U* test, *n* = 4 rats per group. E) The representative protein expression of TREK2 was obtained by western blot in whole DRG cells (upper) and cellular membrane (bottom) lysates derived from the prediabetic rats and control ones. F) The histogram data shows downregulation of TREK2 expression in both whole cells and cellular membranes of the prediabetes group compared to the control one. **p < 0.01, Independent Samples *t*‐test, *n* = 4–5 rats per group. Scale bars: (A), (B), (C) = 100 µm.

Potassium channels represent the largest group of ion channels involved in the modulation of cellular excitability, cell volume, proliferation, and maintenance of K^+^ homeostasis. These channels are divided into four main classes, based on their structure and function: voltage‐gated K^+^ (*K*_v_), Ca^2+^‐activated K^+^ (*K*_Ca_), inwardly rectifying K^+^ (*K*_ir_), and two‐pore domain background K^+^ (*K*_2P_) channels.^[^ ^35^ ^]^ Of these, the *K*_2P_ channel is an important regulator of pain sensation and a potential therapeutic target for pain treatment. To explore the potential mechanism underlying prediabetic pain hypersensitivity, we further analyzed the expression changes of two subunits of the *K*_2P_ family, namely, TREK1 (*K*_2P_2.1) and TREK2 (*K*_2P_10.1) channels, in DRG neurons. These mechanogated and thermosensitive *K* ^+^ channels are crucial for controlling the excitability of somatosensory neurons.^[^ ^36^, ^37^ ^]^ Confocal immunofluorescence analysis revealed decreased expression of TREK1 and TREK2 in NF200‐, IB4‐, and CGRP‐positive neurons (**Figures** **2**A–C and **3**A–C). Grouped data showed that the average immunofluorescence intensities of TREK1 and TREK2 were significantly lower in the prediabetes group than in the control group (Figures 2D and 3D, left panel). The immunofluorescence intensities of TREK1 and TREK2 were analyzed in NF200‐, IB4‐, and CGRP‐positive neurons (Figures 2D and 3D, right panel). The immunofluorescence intensity of TREK1 decreased by more than 54% in both NF200‐ and IB4‐positive neurons, as well as by 34% in CGRP‐positive neurons, as compared to the control group. Meanwhile, the intensity of TREK2 decreased by over 60% in both NF200‐and CGRP‐positive neurons and by 19% in IB4‐positive neurons, as compared to the control group. The percentages of TREK1 and TREK2 expression in NF200‐, IB4‐, and CGRP‐positive neurons were also examined (Figures 2D and 3D, bottom panel). However, there were no significant differences in the cellular types of DRG neurons expressing TREK1 or TREK2 between the prediabetic and control groups (data not shown). To confirm the decreased immunoreactivity of TREK1 and TREK2 observed in prediabetic rats, we extracted crude homogenates from DRGs and analyzed the protein expression of TREK1 and TREK2 using Western blotting. As fully functioning ion channels are transmembrane proteins, we detected the expression of TREK1 and TREK2 in membrane proteins from the DRGs. The quantitative results showed that the expression of these *K*_2P_ channels was significantly decreased in both whole cells and cellular membranes of prediabetic rats (Figures 2E,F and 3E,F).

### 2.4 Mechanical Allodynia and Thermal Hyperalgesia Elicited by the Downregulation of TREK1 and TREK2 through siRNA Intraganglionic Injection

**Figure 4:**
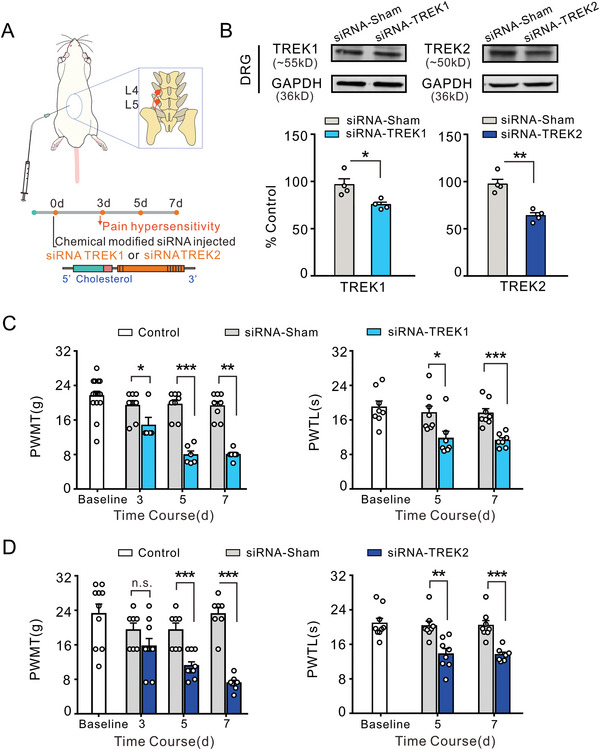
Downregulated the expression of TREK1 and TREK2 induces pain hypersensitization through percutaneous intraganglia siRNA injection. A) Schematic diagram of intraganglia siRNA injection to Sprague‐Dawley (SD) rats. B) The representative immunoblots of TREK1 (upper left) and TREK2 (upper right) were obtained by western blot in DRG lysates derived from the rats injected with sham siRNA and those injected with siRNA. Note that the protein expression of TREK1 (bottom left) and TREK2 (bottom right) was downregulated in the groups injected with siRNA compared to those injected with sham siRNA indicating a significant effect of siRNA on the expression levels of these proteins. *p < 0.05, **p < 0.01, Independent Samples *t*‐test, *n* = 4 rats per group. C) Quantitative analysis shows that PWMT was significantly decreased from the third day following siRNA‐TREK1 injection. The right panel shows that PWTL was not affected by siRNA‐TREK1 until the fifth day after siRNA injection. *p < 0.05, **p < 0.01, ***p < 0.001, Mann Whitney *U* test (left panel) and Independent Samples *t*‐test (right panel), *n* = 5–8 rats per group. D) Quantitative analysis shows a significant decline in PWMT starting from the fifth day after siRNA‐TREK2 injection. Grouped data presented in the right panel indicates that PWTL was significantly decreased commencing from the fifth day after siRNA‐TREK2 injection. **p < 0.01, ***p < 0.001, Independent Samples *t*‐test, *n* = 7–10 rats per group.

TREK1 and TREK2 are mechanogated and temperature‐sensitive K^+^ channels, respectively. To detect whether the downregulation of TREK1/2 could contribute to pain sensitization, siRNA‐TREK1 (50 µm, 20 µL) and siRNA‐TREK2 (50 µm, 20 µL) were percutaneously intraganglionically injected into L4‐L5 DRGs as shown in the schematic diagram (**Figure** **4**A). On the seventh day after TREK1 siRNA injection, western blot data showed a remarkable decrease in TREK1 expression in the DRG neurons as compared to that in the group injected with sham siRNA (Figure 4B, left panel). Similarly, the injection of TREK2 siRNA resulted in a decrease in the expression of TREK2 in the DRG (Figure 4B, right panel). In the pain behavioral test, rats showed mechanical pain hypersensitivity (Figure 4C, left panel) on the third day following siRNA‐TREK1 injection, and up to the fifth day after this siRNA administration, thermal pain hypersensitization occurred (Figure 4C, right panel). Similarly, on the fifth day after the siRNA‐TREK2 injection, the rats showed mechanical (Figure 4D, left panel) and thermal pain hypersensitivity (Figure 4D, right panel). siRNA injections significantly reduced both PWMT and PWTL in the ipsilateral hind paw, whereas no significant changes were observed in the contralateral hindpaws (data not shown). Downregulation of TREK1 or TREK2 expression may result in increased sensitivity to mechanical and thermal pain.

### 2.5 Upregulation of Sortilin Expression in DRG Neurons Following HED, and the Colocalization and Coprecipitation of Sortilin with TREK1 or TREK2

**Figure 5:**
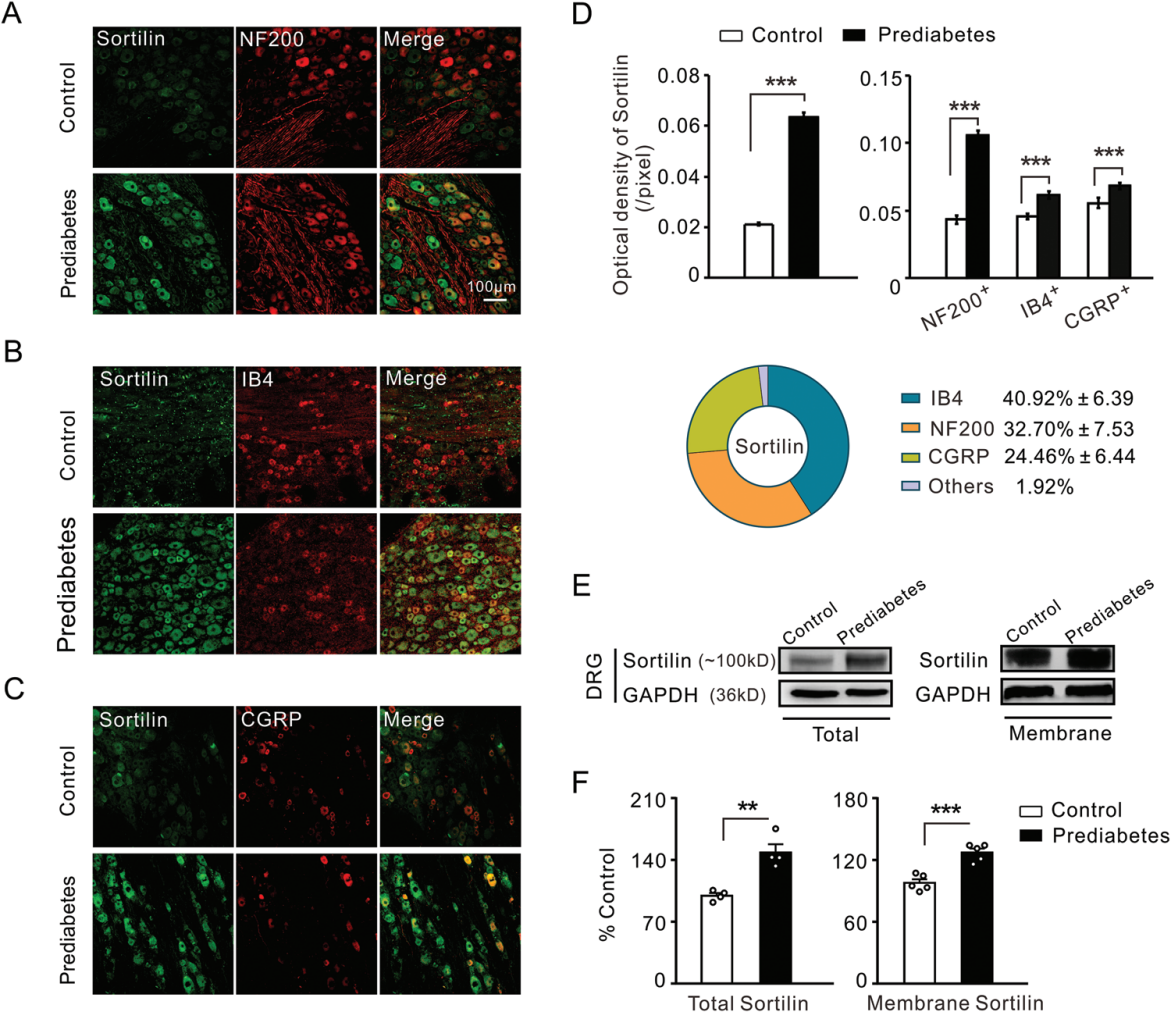
Characterization of the expression profile of Sortilin in DRG neurons reveals that the expression of Sortilin in NF200‐, IB4‐, and CGRP‐positive DRG neurons are increased in prediabetic rats compared to controls. A) Double immunofluorescence staining of DRG neurons with large cell marker NF200 (green) and Sortilin antibody (red) in control rats (top) and prediabetic rats (bottom), X20 magnification. B) Double immunofluorescence staining of DRG neurons with IB4 (green) and Sortilin antibody (red) in control rats (top) and prediabetic rats (bottom), X20 magnification. C) Immunofluorescence labeled of CGRP (green) and Sortilin antibody (red) in DRG neurons from control rats (top) and prediabetic rats (bottom), X20 magnification. D) Quantitative analysis shows that the average immunofluorescence intensity of Sortilin is significantly stronger in the prediabetes group than in the control group (left panel). The right panel shows the immunofluorescence intensity of Sortilin in NF200‐, IB4‐, and CGRP‐positive DRG neurons. The bottom chart shows the percentage of Sortilin immunoreactivity in NF200‐, IB4‐, and CGRP‐positive DRG neurons. ***p < 0.001, Mann Whitney *U* test, *n* = 4 rats per group. E) The expression of Sortilin was obtained by western blot analysis with DRG whole cells (upper) and cellular membrane (bottom) lysates derived from prediabetic and control rats. F) Grouped data in the bottom panel shows that the expression of Sortilin was upregulated in both whole cells and cellular membrane of prediabetes groups compared to controls. **p < 0.01, ***p < 0.001, Independent Samples *t*‐test, *n* = 4–5 rats per group. Scale bars: (A), (B), (C) = 100 µm.

In recent years, Sortilin has gained considerable attention for its functions in intracellular trafficking, sorting, and cell surface receptor internalization, as well as its role in regulating lipid metabolism.^[^ ^33^ ^]^ Sortilin facilitates the trafficking of TREK1 from the hippocampal neuronal membrane to the lysosomes, thereby playing an important role in regulating TREK1 expression.^[^ ^34^ ^]^ To investigate whether Sortilin regulates the expression of TREK1 and TREK2 in DRG neurons in prediabetic conditions, we examined the expression of Sortilin in DRG neurons from both prediabetic and control rats, as well as confirmed its interaction with these TREK channels. In the present study, increased Sortilin expression was observed in NF200‐, IB4‐, and CGRP‐positive neurons (**Figure** **5**A–C). Quantitative analysis showed that the average immunofluorescence intensity of Sortilin was significantly increased in the prediabetes groups as compared to controls (Figure 5D, left panel). The immunofluorescence intensity of Sortilin was analyzed in NF200‐, IB4‐, and CGRP‐positive neurons (Figure 5D, right panel). The immunofluorescence intensity of Sortilin significantly increased by more than 145% in NF200‐positive neurons and by 35% in IB4‐positive neurons as compared to the control group. Furthermore, the percentages of Sortilin expression in NF200‐, IB4‐, and CGRP‐positive neurons were evaluated separately (Figure 5D). Consistent with the changes observed in the immunofluorescence test, Sortilin overexpression was detected by western blot analysis of DRG lysates derived from prediabetic rats in comparison with control rats (Figure 5E, left panel). The induction of endocytosis and transportation of TREK1 protein to lysosomes relies on its binding to Sortilin in the cellular membrane. Therefore, we detected Sortilin expression in the membrane proteins of the DRGs. Quantitative results revealed that the expression of Sortilin in the cellular membrane was significantly increased in prediabetic rats as compared to that in control rats (Figure 5E,F, right panel).

**Figure 6:**
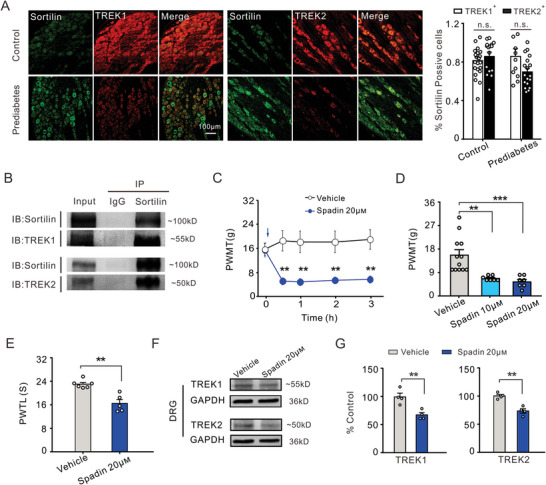
Protein interaction and co‐localization of Sortilin with TREK1 and TREK2. A) The immunofluorescence of Sortilin (green) co‐expressed with TREK1 (red) in both control (top) and prediabetic rats (bottom), X20 magnification. The middle panel shows that immunofluorescence of Sortilin (green) co‐expressed with TREK2 (red) in both control (top) and prediabetic rats (bottom), X20 magnification. The histogram data shows the percentage of Sortilin immunoreactivity in TREK1‐ and TREK2‐positive DRG neurons. n.s., no significance, Mann Whitney *U* test (control group) and Independent Samples *t*‐test (prediabetes group), *n* = 4 rats per group. B) Representative immunoblots depicting the co‐immunoprecipitation of Sortilin with TREK1 and TREK2 in DRG lysates derived from prediabetic rats. C) The time course curves show that PWMT significantly decreased from half an hour after 20 µm Spadin injection (*n* = 6). *p < 0.05, Two‐way ANOVA RM, *n* = 6–7 rats per group. D) The histogram indicates a dose‐dependent decrease in PWMT of rats after 10 and 20 µm Spadin injection. **p < 0.01, Spadin 10 µm versus vehicle, ***p < 0.001, Spadin 20 µm versus vehicle, Kruskal–Wallis with Bonferroni post hoc correction, *n* = 7–12 rats per group. E) PWTL was reduced by 20 µm Spadin treatment. **p < 0.01, Independent Samples *t*‐test, *n* = 7–12 rats per group. F) The representative immunoreactive bands of TREK1 (top) and TREK2 (bottom) were respectively detected by western blot analysis in DRG lysates derived from the vehicle group and Spadin‐injected group. G) The histogrammic data indicates that the expression of TREK1 and TREK2 were suppressed in rats treated with Spadin compared to those treated with vehicle (*n* = 4 vs 4). **p < 0.01, Independent Samples *t*‐test, *n* = 4 rats per group.

To detect the functional interaction between Sortilin and the *K*_2P_ potassium channel, we performed double immunofluorescence and co‐immunoprecipitation assays to investigate whether Sortilin interacts with TREK1 and TREK2. Double staining showed that Sortilin extensively co‐localized with TREK1 and TREK2 in DRG neurons from both control and prediabetic rats (**Figure** **6**A). The proportion of Sortilin expressed in neurons labeled with TREK1 or TREK2 was subsequently analyzed. In the control group, 81.5% of TREK1‐labeled neurons and 86.2% of TREK2‐labeled neurons co‐expressed Sortilin. In the prediabetic group, 86% of TREK1‐labeled neurons and 69.8% of TREK2‐labeled neurons co‐expressed Sortilin (Figure 6A, right panel). Furthermore, co‐immunoprecipitation assays were performed to further elucidate the association between Sortilin and TREK1/TREK2. Sortilin co‐precipitated with both TREK1 and TREK2 in the DRG lysates derived from prediabetic and control rats (Figure 6B).

Spadin, a propeptide derived from Sortilin, exerts a partial inhibitory effect on TREK1 channels in order to maintain the balance of TREK1 expression at physiological concentrations. However, a substantial quantity of Spadin was sufficient to internalize the Sortilin‐TREK1 complex and subsequently suppress the expression of TREK1.^[^ ^34^ ^]^ Therefore, the administration of Spadin partially reproduced the effect of overexpressed Sortilin on TREK channels. In the present study, we found that the recombinant Spadin peptide suppressed the expression of both TREK1 and TREK2, leading to pain hypersensitivity. 30 min after percutaneous intraganglionic injection of Spadin in the L4‐L5 DRGs, rats exhibited a dramatic decline in PWMT as compared to those injected with the vehicle (saline) (Figure 6C). Interestingly, testing the effects of different concentrations of Spadin on PWMT revealed that its algogenic effect was dose‐dependent (Figure 6D). Similarly, Spadin injection (20 µm) elicited thermal pain sensitization as evidenced by a significant decrease in PWTL (Figure 6E), with a concomitant reduction of 28.47% in PWTL observed upon Spadin administration. To further verify the inhibitory effect of Spadin on the expression of TREK1 and TREK2, western blot analysis was performed using DRG lysates derived from both the Spadin‐injected and vehicle groups. The expression of both TREK1 and TREK2 was significantly reduced in response to this injection (Figure 6F,G). However, the mRNA levels of TREK1 and TREK2 remained unaltered following Spadin administration (Figure S3, Supporting Information).

### 2.6 The Regulation of Sortilin on TREK1 and TREK2 Expression

**Figure 7:**
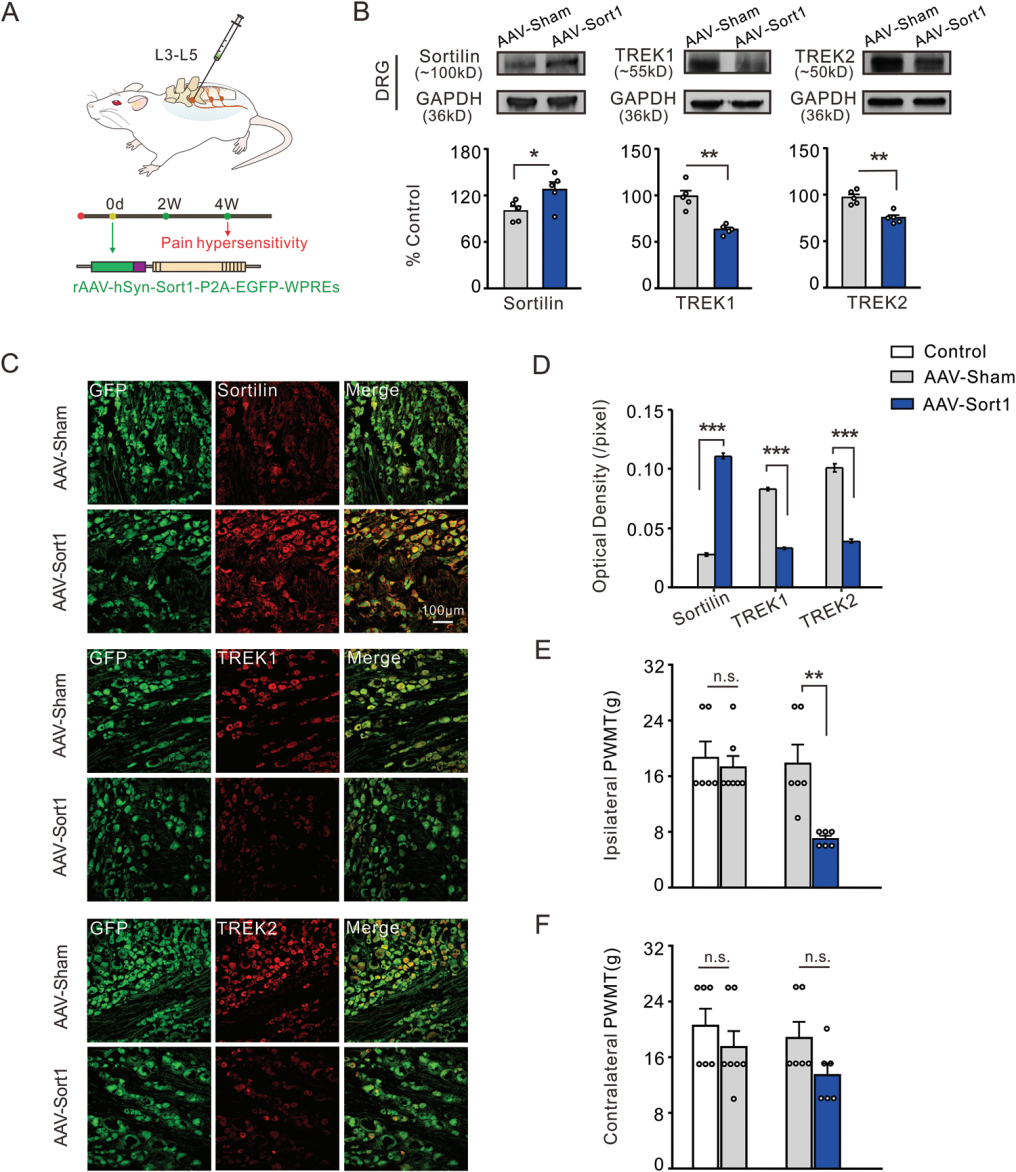
Intra‐DRG AAV‐Sort1 injection elicits pain hypersensitivity and downregulates the expression of TREK1 and TREK2. A) Schematic diagram of intra‐DRG virus injected in naïve SD rats. B) The representative immunoblots of Sortilin (upper left), TREK1 (upper middle), and TREK2 (upper right) were obtained by western blot in DRG lysates derived from the AAV‐sham injected rats and AAV‐Sort1 injected rats. Quantitative analysis shows that the expression of Sortilin was increased, while TREK1 and TREK2 were decreased in the AAV‐Sort1 injection group compared to the AAV‐sham injection group. *p < 0.05, **p < 0.01, Independent Samples *t*‐test, *n* = 5 rats per group. C) Double immunofluorescence staining demonstrates an effective intervention in the expression of Sortilin (upper red), TREK1 (middle red), and TREK2 (bottom red) in DRG neurons by AAV‐Sort1 treatment, X20 magnification. D) The histogram shows that the average immunofluorescence intensity of Sortilin significantly increased, while TREK1 and TREK2 decreased in the AAV‐Sort1 treatment group compared to the AAV‐sham treatment group. ***p < 0.001, AAV‐Sort1 versus AAV‐Sham, Mann Whitney *U* test, *n* = 4 rats per group. E,F) The quantitative data on mechanical threshold indicates that Sortilin overexpression in DRG cells exacerbated mechanical pain sensitivity on the ipsilateral side but not on the contralateral side. n.s., no significance, AAV Sham versus control, AAV Sort1 versus AAV Sham, **p < 0.01, AAV Sort1 versus AAV Sham, Mann Whitney *U* test, *n* = 6–7 rats per group.

Short peptides derived from Sortilin inhibit the expression of TREK1 and TREK2, thereby inducing pain hypersensitivity. Next, to investigate whether the expression of TREK1 and TREK2 is directly regulated by Sortilin in DRG neurons and to explore the potential involvement of overexpressed Sortilin protein in the negative regulation of TREK1 and TREK2, *r*AAV‐hSyn‐Sort1‐P2A‐EGFP‐WPREs was intra‐DRG injected to upregulate the expression of Sortilin in normal rats, as illustrated schematically (**Figure** **7**A). The expression levels of TREK1/TREK2 channels and Sortilin in the DRG samples were examined. Crude homogenates of the DRGs were extracted, followed by western blot analysis to determine the protein expression levels of Sortilin, TREK1, and TREK2. The quantitative results indicated a significant reduction in both TREK1 and TREK2, but an increase in Sortilin in AAV‐Sort1 transfected rats as compared to sham AAV‐injected rats (Figure 7B). Meanwhile, in order to further support the immunoblots observed in AAV‐Sort1 transfected rats, double immunofluorescence experiments were performed using confocal microscopy. The results revealed a significant decrease in the immunoreactive intensity of TREK1 and TREK2, as well as an increase in the immunofluorescence intensity of Sortilin within AAV‐Sort1 injected groups (Figure 7C,D). Furthermore, these genetically modified rats exhibited nociceptive behavior owing to the upregulation of Sortilin expression in DRG neurons. Intra‐DRG injection of AAV‐Sort1 induced pain hypersensitivity in normal rats. In contrast to sham AAV injection, the PWMT of the ipsilateral hindpaws in rats was significantly decreased in the fourth week following intra‐DRG injection of AAV‐Sort1 (Figure 7E), but there was no significant change in the values of contralateral hindpaws (Figure 7F). These data suggest that Sortilin overexpression is associated with pain sensitization due to the negative regulation of TREK1/TREK2 expression.

**Figure 8:**
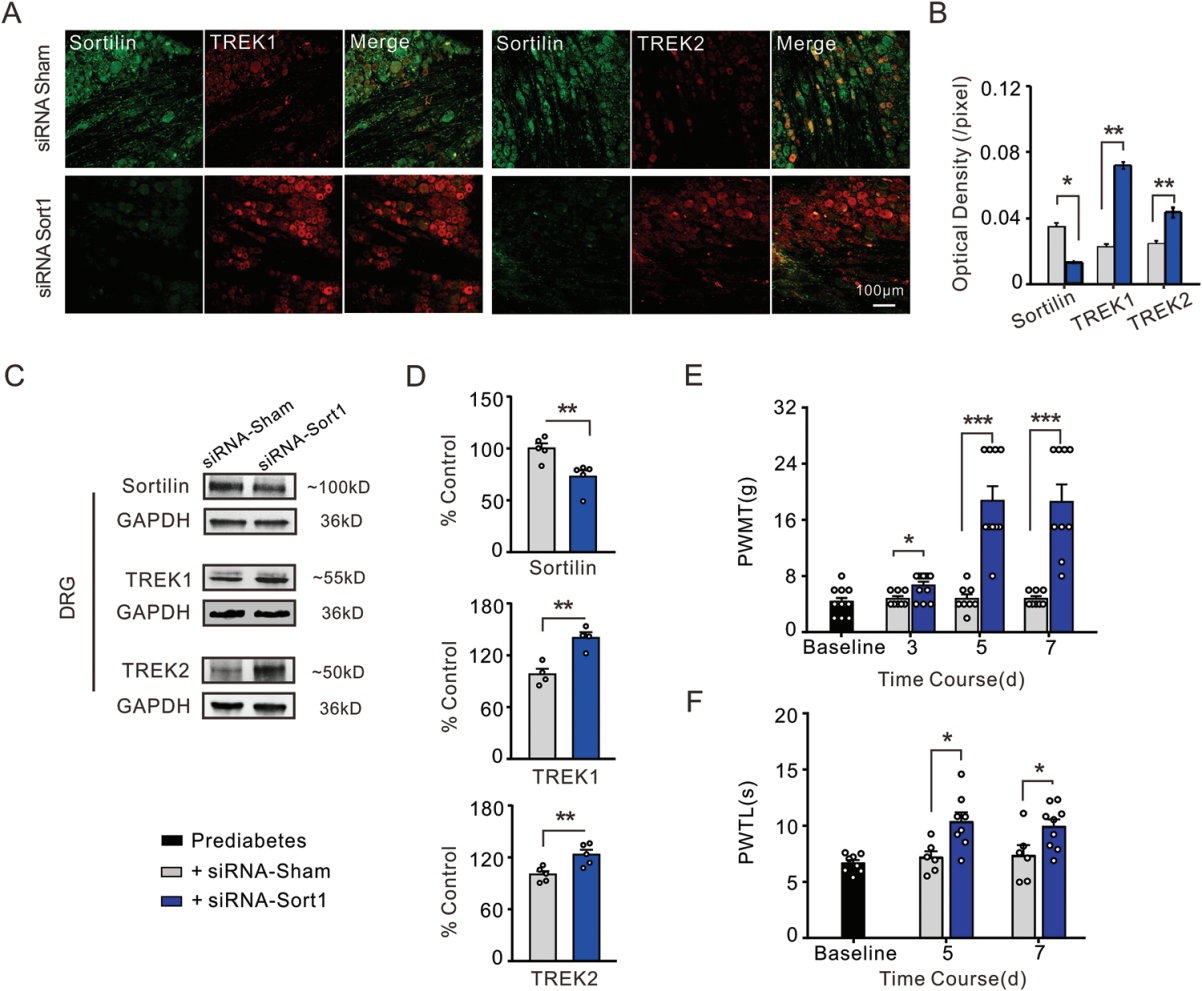
The percutaneous intraganglia injection of siRNA targeting Sortilin in prediabetic rats resulted in a reduction of pain hypersensitization and an upregulation of TREK1 and TREK2 expression. A) Double immunofluorescence staining of DRG neurons with Sortilin (green) and TREK1 antibody (red) from rats injected with sham siRNA (top) and Sortilin siRNA (bottom), X20 magnification. The right panel shows the immunofluorescence staining of Sortilin (green) and TREK2 antibody (red) in rats injected with sham siRNA (top) and Sortilin siRNA (bottom), X20 magnification. B) Quantitative analysis shows that the average immunofluorescence intensity of Sortilin significantly decreased in the siRNA‐injected group compared to the sham‐injected group. The intensity of TREK1 and TREK2 significantly increased in DRG neurons from the rats injected with Sortilin siRNA compared to those injected with sham siRNA. ***p < 0.001, siRNA‐Sort1 versus siRNA‐Sham, Mann Whitney *U* test, *n* = 4 rats per group. C) The representative immunoblots show efficient knockdown of Sortilin and upregulated expression of TREK1 and TREK2 in DRG from prediabetic rats. D) Quantitative summary of levels of Sortilin, TREK1, and TREK2 in DRG lysates from rats injected with sham siRNA and those injected with Sortilin siRNA. **p < 0.01, siRNA‐Sort1 versus siRNA‐Sham, Independent Samples *t*‐test, *n* = 5 rats per group. E) The grouped data show that the PWMT was significantly increased from the third day after Sortilin siRNA injection compared with sham siRNA injection. *p < 0.05, ***p < 0.001, siRNA‐Sort1 versus siRNA‐Sham, Mann Whitney *U* test, *n* = 8–12 rats per group. F) In contrast to the sham siRNA‐injected group, the PWTL (bottom panel) was markedly attenuated in the Sortilin siRNA‐treated group on the fifth day following siRNA injection. *p < 0.05, siRNA‐Sort1 versus siRNA‐Sham, Independent Samples *t*‐test, *n* = 6–9 rats per group.

To further elucidate the regulatory role of Sortilin in these TREK channels, we reversed Sortilin overexpression in DRG neurons of PDNP rats using a genetic intervention approach. siRNA‐Sort1 (50 µm, 20 µL) was used to knock down Sortilin. Percutaneous intraganglionic injection of siRNA‐Sort1 decreased Sortilin expression but enhanced TREK1 and TREK2 expression, as detected by double immunofluorescence staining and western blotting. Despite the decreased immunofluorescence intensity of Sortilin, the intensities of TREK1 and TREK2 were significantly elevated in the siRNA‐Sort1 injection group as compared to those in the siRNA‐sham injection group (**Figure** **8**A,B). Further analysis was performed on the expression of Sortilin, TREK1, and TREK2 by western blotting (Figure 8C). Immunoblot analysis of DRG cell lysates revealed that Sortilin knockdown increased the expression of TREK1 and TREK2. Quantitative data also demonstrated that the siRNA‐Sort1 injection efficiently decreased the expression of Sortilin and increased the expression of TREK1 and TREK2 (Figure 8D). In addition, percutaneous intraganglionic injection of siRNA targeting Sortilin significantly attenuated pain sensitization in prediabetic rats. Compared to the sham siRNA injection, there was a significant increase in PWMT values in the ipsilateral hindpaws on the fifth day after siRNA‐Sort1 injection (Figure 8E). Similarly, thermal pain sensitivity was alleviated in the ipsilateral hindpaws on the fifth day after siRNA‐Sort1 injection as compared to that after siRNA‐sham injection (Figure 8F).

### 2.7 Decreased Potassium Current Mediated by TREK1 and TREK2 in Prediabetic DRG Neurons

**Figure 9:**
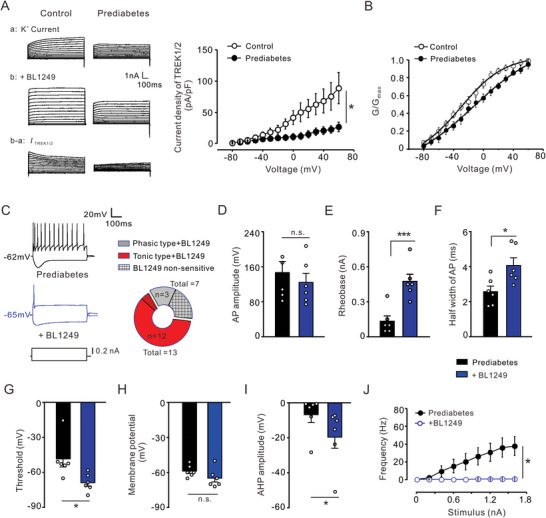
A smaller *I*_TREK1/2_ is detected in prediabetic neurons, and the administration of BL‐1249, a TREK1/2 channel opener, effectively attenuates neuronal hyperexcitability in prediabetic rats. A) Representative traces of *I*_TREK1/2_ were evoked by 700 ms depolarizing commands ranging from −80 to 50 mV. These panels show total *K*v current (a), potassium current with BL1249 treatment (b), and *I*_TREK1/2_ (c = b − a), respectively. The holding potential was held at −80 mV. The total *K*v current (a) was recorded following a 1s prepulse to −120 mV. *I*_TREK1/2_ is the difference current between (b) and (a). *I–V* relations curves of *I*_TREK1/2_ were obtained from control and prediabetic groups (*n* = 13 vs 7). The amplitudes of *I*_TREK1/2_ in DRG neurons from prediabetic rats were greatly decreased under depolarized stimuli. *p < 0.05, **p < 0.01, ***p < 0.001, Two‐way ANOVA RM, *n* = 7 cells for prediabetes versus 15 cells for control. B) The voltage‐dependent activation curves of *I*_TREK1/2_ were evaluated from the prediabetic (*n* = 7, filled circles) and control (*n* = 15, opened circles) group. C) Representative traces (left upper) showing the spike firings in response to a depolarizing current step in DRG neurons from the prediabetic group and the prediabetic + BL1249 group. The protocol for recording the action potentials is shown at the bottom. The circular chart shows the distribution of BL1249‐sensitive neurons in cells with tonic firing type and phasic firing type, respectively. D) The histogram indicates that there was no significant change in the amplitude of action potential between pre and post‐BL1249 treatment. n.s., no significance, Paired samples *t*‐test, *n* = 6 cells per group. E) Grouped data show that the rheobase for the first action potential firing increased between pre and post‐BL1249 treatment (*n* = 6). ***p < 0.001, Paired samples *t*‐test, *n* = 6 cells per group. F) Treatment with BL1249 prolonged the half‐width of action potential. *p < 0.05, Paired samples *t*‐test, *n* = 6 cells per group. G) The threshold for action potential exhibited greater depolarization after BL1249 treatment. *p < 0.05, Paired Samples Wilcoxon Signed Rank Test, *n* = 6 cells per group. H) The membrane potential had no statistical difference between pre‐ and post‐BL1249 treatment. n.s., no significance, Paired samples *t*‐test, *n* = 6 cells per group. I) This panel indicated that the peak amplitude of after hyperpolarization potential enlarged after BL1249 treatment. *p < 0.05, Paired Samples Wilcoxon Signed Rank Test, *n* = 6 cells per group. J) The plots show that firing frequency was notably decreased in the BL1249 treated group compared to the vehicle group. *p < 0.05, **p < 0.01, Two‐way ANOVA RM, *n* = 9 cells per group.

In the nervous system, TREK1 and TREK2 channels contribute to hyperpolarization of the RMP, acceleration of action potential repolarization, and suppression of electrical activity.^[^ ^38^ ^]^ In order to understand the changes in potassium currents mediated by TREK1 and TREK2 channels (*I*_TRKE1/2_) under painful prediabetic conditions, further investigation is necessary. Therefore, the current study detected the magnitude and impact of these *K*_2P_ currents in DRG neurons of PDNP rats. To examine the magnitude of *I*_TRKE1/2_, we isolated *I*_TRKE1/2_ using a pharmacological approach. *K*v current was elicited under a voltage clamp by depolarizing the potentials, as mentioned above (**Figure** **9**Aa). Following this, the bath application of BL1249, a selective TREK1/2 channel opener, activated the TREK1/2 channels to induce an amplified *K*v current (Figure 9Ab). Pure *I*_TRKE1/2_ was obtained by digitally subtracting the *K*v current from the amplified *K*v current (Figure 9Ac). Compared with the control groups, the current density of *I*_TREK1/2_ was noticeably decreased in DRG neurons in the prediabetic groups (Figure 9A, right panel). The *V*_1/2_ of activation for *I*_TREK1/2_ was shifted in the depolarizing direction in prediabetic neurons. This *V*_1/2_ value was approximately −11.96 ± 0.28 mV in the prediabetic groups which was at a more positive voltage than in control groups (−28.36 ± 0.24 mV, Figure 9B). Collectively, these findings suggest that a reduction in *I*_TREK1/2_ may contribute to the neuronal hyperexcitability induced by PDNP.

### 2.8 Alleviation of Neuronal Hyperexcitability and Painful Sensitivity in Prediabetic Rats via Treatment with a TREK1/TREK2 Agonist

We further tested the modulation of neuronal excitability by TREK1 and TREK2 channels using a pharmacological approach. The bath application of BL1249 (10 µm) significantly decreased the excitability of DRG neurons from prediabetic animals. Notably, this agonist nearly abolished repeated firing in prediabetic neurons (Figure 9C, left panel). In prediabetic DRG, 92.3% of tonic firing type neurons responded to BL1249 in the current clamp recording, whereas only 42.8% of phasic firing type cells were sensitive to BL1249 (Figure 9C, right panel). The amplitude of the action potential was unaffected by the BL1249 treatment (Figure 9D). However, the application of BL1249 significantly increased both the rheobase (Figure 9E) and half‐width (Figure 9F) of the action potential. TREK1/2 channels are capable of maintaining cellular membrane hyperpolarization below the action potential threshold. Therefore, the pharmacological activation of TREK1/2 channels by BL1249 further hyperpolarized the threshold voltage of the action potential in prediabetic DRG neurons (Figure 9G). The bath application of BL1249 markedly enhanced the amplitude of AHP in prediabetic DRG neurons (Figure 9I). However, passive membrane properties of prediabetic DRG neurons, such as membrane potential and capacitance (data not shown), remained unchanged after the BL1249 application (Figure 9H). More importantly, the mean firing frequency induced by a depolarizing current stimulation was much lower in the treated BL1249 neurons (Figure 9J). In addition, neuronal TREK1 and TREK2 channels regulate mechanosensitivity and thermosensitivity to exert analgesic and neuroprotective effects. In the present study, BL1249 (10 µm, 20 µL) was percutaneously intraganglionically injected into L4‐L5 DRGs to evaluate the analgesic effect resulting from TREK1/2 activation. Pain behavioral tests were performed, and the ipsilateral PWMT and PWTL values of these rats were observed to significantly increase starting from 90 min after BL1249 injection (**Figure** S4A,B, Supporting Information).

**Figure 10:**
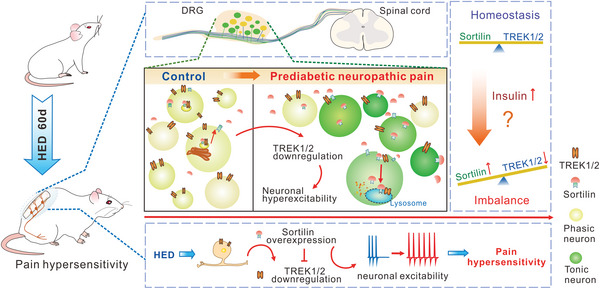
The schematic shows that overexpression of Sortilin leads to a downregulation in the expression of TREK1 and TREK2, resulting in neuronal hyperexcitability of DRG and persistent pain hypersensitivity of HED rats. Rats on a HED develop prediabetic neuropathic pain despite the absence of hyperglycemia. Contrary to the normal chow group, DRG neurons from the HED‐fed rat exhibit tonic firing and hyperexcitability due to the decreased expression of TREK1 and TREK2. Sortilin, an overexpressed sorting protein in DRG neurons of the HED group, plays an important role in trafficking TREK1 and TREK2 proteins. Thereby, overexpression of Sortilin downregulates the expression of TREK1 and TREK2 channels in DRG neurons, resulting in neuronal hyperexcitability and severe neuropathic pain.

## 3 Discussion

The present study elucidated the role of TREK1 and TREK2 in regulating prediabetic nociceptive sensitization and further demonstrated that overexpression of Sortilin could negatively regulate the expression of TREK1 and TREK2 in peripheral sensory neurons associated with PDNP. We discovered a significant upregulation of Sortilin in the DRG neurons of rats with PDNP, where it interacted with the TREK1 and TREK2 channels. This upregulation of Sortilin was found to inhibit the expression of TREK1 and TREK2 channels via internalization in the cell membrane. Consequently, in prediabetic rats, pain hypersensitivity and sensory neuronal hyperexcitability may be caused by Sortilin overexpression‐mediated downregulation of TREK1/2 channels in DRG neurons, which represents a potential new mechanism underlying PDNP (Figure 10).

### 3.1 Involvement of Peripheral Neuronal Hyperexcitability and Downregulated K^+^ Current in PDNP

Diabetic neuropathic pain is a prevalent complication of diabetes, and its mechanism has been extensively investigated. However, with the concepts of prediabetes and PDN being proposed, numerous studies have reported that pain symptoms can occur at this stage. However, the precise molecular mechanisms underlying these processes remain unknown. In the present study, we observed significant mechanical allodynia and thermal hyperalgesia in rats with HED‐induced prediabetes. Furthermore, we assessed functional alterations in primary afferent neurons of rats treated with PDNP. The DRG neurons of these animals exhibited hyperexcitability including enhanced action potential amplitude, firing frequency, and reduced firing threshold. Although the underlying mechanisms are different in these cases, similar hyperexcitability has also been observed in other animal models of pain, such as streptozotocin (STZ)‐induced diabetic neuropathic pain; inflammatory pain induced by injection of complete Freund's adjuvant (CFA), carrageenan, formalin, or bee venom; and spared nerve injury (SNI)‐induced neuropathic pain.^[^ ^39^, ^40^, ^41^, ^42^ ^]^ Neuronal excitability is partially determined by the activity of ion channels. Na^+^ and K^+^ channels are directly involved in eliciting action potentials. Additionally, many previous studies have revealed a clear role of K^+^ channels in neuronal excitability and pain perception in diverse painful peripheral neuropathies. For example, A‐type K^+^ currents (carried by Kv1.4, Kv3.4, Kv4.1, Kv4.2, and Kv4.3 subunits) and dendrotoxin (DTX)‐sensitive K^+^ currents (carried by Kv1.1, Kv1.2, and Kv1.6 subunits), which regulate neuronal hyperexcitability, were decreased in DRG neurons derived from rats with diabetic neuropathic pain or sciatic nerve axotomy.^[^ ^43^, ^44^ ^]^ Furthermore, *K*_Ca_ current (carried by BK, IK, and SK channels) was reduced in the DRG neurons of rats with SNL,^[^ ^45^, ^46^, ^47^ ^]^ and a deficiency in the BK channel was also observed in pain models induced by SNI and persistent inflammation.^[^ ^48^, ^49^ ^]^ Additionally, *K*_2P_‐induced leak currents (carried by TESK, TASK2, and TREK‐1 channels), which are crucial determinants of neuronal repetitive firing and nociceptive processing, were reduced in CFA‐ and carrageenan‐induced inflammatory pain and nerve injury‐induced neuropathic pain.^[^ ^20^, ^50^ ^]^ However, it remains unknown whether these K^+^ channels contribute to the effects of PDNP. To clarify the mechanism underlying PDNP‐induced hyperexcitability, we evaluated K^+^ currents. A significantly decreased total K^+^ current was detected in PDNP rats. Furthermore, we focused on *K*_2P_ channels, which are an important component of the K^+^ channel family that modulates membrane potential, influences the action potential threshold, and affects the firing frequency of DRG neurons.

However, the involvement of Na^+^ and other K^+^ channels in the regulation of neuronal hyperexcitability, especially regarding action potential threshold, AHP, and repetitive firing, cannot be disregarded in PDNP models, although these channels were not specifically addressed in the current study. For instance, the Na^+^ channel subtype Nav1.7 is exclusively expressed in the nociceptive pathways of the PNS and regulates the action potential threshold, which causes an enlarged persistent sodium current associated with SNI‐induced neuropathic pain, STZ‐induced diabetic neuropathic pain, and inflammatory pain.^[^ ^39^, ^51^, ^52^ ^]^ *K*_Ca_ channels modulate neuronal firing frequency and AHP, generating an outward current that prevents neuronal hyperexcitability.^[^ ^53^, ^54^ ^]^ However, these channels have not been thoroughly studied in the context of PDNP. Future studies should examine their involvement in PDNP in greater detail.

### 3.2 Downregulation of TREK1 and TREK2 in DRG Neurons and their Contribution to PDNP

The TREK subfamily, which consists of three channels—TREK1, TREK2, and TRAAK—represents a subdivision of *K*_2P_ channels that are prominently expressed in the nervous system and play crucial roles in controlling neuronal excitability and modulating nociception. This subfamily is widely expressed in the PNS and is activated by lipids, temperature, and mechanical stimuli. Within this subfamily, TREK1 exhibits a 63% sequence identity with TREK2, while the identity decreases to 45% with TRAAK. These three channels interact synergistically by forming heterodimers. Notably, there is a higher affinity observed between TREK1 and TREK2 (60 ± 8%) compared to that between TRAAK and TREK1 (50.8 ± 6.6%) or TREK2.^[^ ^55^ ^]^ Importantly, the background K^+^ currents are primarily produced by the TREK2 channel (69%), followed by the TREK1 (12%) and TRAAK (3%) channels.^[^ ^22^ ^]^ Therefore, the current study highlighted the functional roles of TREK1 and TREK2 in PDNP.

Immunostaining of TREK1 and TREK2 in the DRG demonstrated that TREK channels distributed in CGRP‐labeled peptidergic and IB_4_‐labeled nonpeptidergic small‐ and medium‐sized neurons. This is consistent with numerous previous studies showing that TREK1 channels are predominantly expressed in small‐ and medium‐sized DRG neurons, whereas TREK2 channels are expressed in IB_4_‐labeled neurons.^[^ ^50^ ^]^ Recent research, however, has indicated that TREK1 and TRAAK channels may be highly expressed at Ranvier's node of mammalian myelinated Aβ‐afferent nerves.^[^ ^56^, ^57^ ^]^ Based on this, we wanted to detect whether the TREK channels were present in large‐sized DRG neurons, as these channel proteins are synthesized within the neuronal soma and then transported to the axons for axoplasmic transport. Interestingly, our present observations confirmed this assumption and showed that TREK1 and TREK2 co‐localize with NF200, a selective marker for larger‐sized neurons.

According to literature reviews, TREK1, an extensively investigated *K*_2P_ channel, plays a crucial role in neuronal thermo‐ and mechano‐activation.^[^ ^17^, ^18^, ^58^ ^]^ TREK1‐deficient mice exhibit increased sensitivity to thermal and mechanical stimuli, indicating that TREK1 functions as a signal integrator in response to physiological and pathological stimuli. Additionally, the reduction in TREK1 channels contributes to the development of mechanical and thermal hypersensitivity in individuals experiencing persistent inflammatory and neuropathic pain, such as CFA‐, carrageenan‐induced inflammatory pain, and oxaliplatin treatment‐induced neuropathic pain.^[^ ^24^, ^36^, ^59^ ^]^ In addition to TREK1, TREK2 is also a *K*_2P_ channel sensitive to both thermal and mechanical stimuli. Both TREK1 and TREK2 channels are expressed in the PNS and CNS, whereas expression of the TREK2 channel is relatively low in the CNS.^[^ ^60^ ^]^ Intriguingly, the TREK2 channel contributes to limiting spontaneous pain behavior in CFA‐induced inflammatory pain; however, it remains unknown whether the TREK1 channel serves a similar function.^[^ ^50^ ^]^ Furthermore, these channels exhibit distinct thermal activation thresholds, for instance, the activity of TREK1 current increases 20‐fold when temperature rises from 22 to 42 °C, whereas maximal activation of TREK2 is observed near 37 °C.^[^ ^61^ ^]^ Nevertheless, deficiency in either TREK1 or TREK2 in mice can result in comparable mechanical allodynia and thermal hyperalgesia. According to Royal et al., TREK1 and TREK2 also serve as potential molecular targets for understanding migraine. The inhibition of both TREK1 and TREK2 channels may induce hyperexcitability in trigeminal sensory neurons, leading to migraine‐like symptoms in rodents.^[^ ^25^ ^]^ Additionally, visceral inflammation could increase neuronal mechanosensitivity by reducing the expression of TREK1 and TREK2 mRNA in DRG neurons.^[^ ^62^ ^]^ Given the established importance of TREK1 and TREK2 in nociception, we investigated the expression and functionality of these channels in peripheral sensory neurons in the context of our PDNP study. Our current findings showed that the expression of TREK1 and TREK2 was markedly downregulated in DRG neurons of PDNP rats. In addition, we mimicked this downregulation in normal rats. It would be a captivating pursuit to investigate the individual impact of TREK1 or TREK2 on nociceptive thermo‐ and mechanosensitivity in PDN. In the present study, precise knockdown of either TREK1 or TREK2 in the lumbar DRG resulted from intraganglion‐injected siRNAs. When siRNA‐TREK1 was used to suppress TREK1 expression, TREK2 protein levels did not decrease and vice versa (Figure S5, Supporting Information). Furthermore, with siRNA was administered to the DRG of normal rats to genetically inactivate TREK1 or TREK2, these rats displayed severe mechanical allodynia and thermal hyperalgesia. The findings elucidate the crucial roles of TREK1 and TREK2 in pain development in PDN. These findings lead to the conclusion that TREK1 and TREK2 channels in DRG neurons play crucial roles in regulating mechanical allodynia and thermal hyperalgesia in PDN, which suggests that these channel proteins are potential therapeutic targets for managing PDNP.

### 3.3 Contribution of Sortilin‐Mediated Inhibition of TREK1/2 Channels in DRG Neurons to PDNP

Although a dramatic change in the expression of TREK1 and TREK2 occurs in DRG neurons in the PDNP state, the molecular mechanism underlying this change is still unknown. A variety of molecular regulators govern the function of TREK channels.^[^ ^63^ ^]^ A scaffolding protein called AKAP150, which is known to organize signaling complexes in neurons, can bind to the region between V298 and R311 in the TREK1 key regulatory domain, potentially speeding up the inactivation of TREK1. Additionally, this protein interacts with the highly homologous regulatory domain of TREK2, but not with TRAAK.^[^ ^30^ ^]^ Microtubule‐associated protein 2 has also been identified as a TREK channel partner by the proteomic method (Mtap2, also known as MAP2). Both the TREK1 and TREK2 channels, but not TRAAK, had a conserved eight‐amino acid segment to bind to Mtap2. Furthermore, Mtap2 does not change the gating properties of the TREK channel but increases channel density at the plasma membrane.^[^ ^31^ ^]^ β‐COP, a subunit of coatomer protein complex 1 (COP1), was identified as a direct interacting partner of TREK1, and they colocalized at the membrane. β‐COP could enhance the anterograde transport of TREK1 to the membrane.^[^ ^32^ ^]^ Sortilin is another accomplice in TREK1 trafficking. This protein bidirectionally regulates TREK1 membrane expression. Mazella et al. found that Sortilin directly binds to TREK1 in the Golgi apparatus, thereby promoting TREK1 trafficking to the plasma membrane of mouse cortical neurons. Spadin, a propeptide released from the extracellular N‐terminal domain of Sortilin, binds directly to the TREK1/Sortilin complex and subsequently causes TREK‐1 endocytosis from the membrane. In the CNS, this peptide is regarded as a potential therapeutic endogenous antidepressant.^[^ ^34^ ^]^ According to available literature, Sortilin has been implicated in the pathogenesis of diabetes and metabolic disorders, and its regulation by insulin has been suggested. It is upregulated in the brains of patients with Alzheimer's disease and the serum of patients with diabetes.^[^ ^33^, ^64^, ^65^, ^66^ ^]^ This protein is also associated with the regulation of inflammatory and neuropathic pain by gating BDNF signaling. Sortilin‐mediated BDNF secretion from DRG neurons is involved in CFA‐induced inflammatory pain. Reduction of Sortilin attenuates the BDNF‐induced decrease in potassium chloride cotransporter 2 (KCC2) levels in spinal dorsal horn neurons and alleviates SNI‐induced neuropathic pain. Therefore, disruption of the Sortilin‐BDNF interaction reduces BDNF release and may serve as a therapeutic approach for chronic pain. Furthermore, Sortilin upregulation was also observed in CFA‐induced inflammatory pain, although transgenic Sort1^−^/^−^ mice exhibited normal responses to acute mechanical and thermal stimuli.^[^ ^67^, ^68^ ^]^ In the present study, we found that Sortilin was overexpressed and regulated the membrane expression of TREK1 and TREK2 channels in DRG neurons under PDNP conditions. Furthermore, we explored the molecular modulatory mechanism underlying the decreased expression of TREK1 and TREK2 in DRG neurons in PDNP. An immunoprecipitation‐based proteomic approach detected Sortilin as a direct interacting partner of the TREK1 and TREK2 channels in DRG neurons. Furthermore, the genetic knockdown of Sortilin increased the expression of TREK1 and TREK2 in DRG neurons and vice versa. Unfortunately, the potential role of insulin as an inducer of Sortilin overexpression in the HED‐feeding rats was not investigated in this study. Moreover, further research is needed to determine whether Sortilin interacts with TREK1 or TREK2 alone or with the TREK1/TREK2 complex.

### 3.4 Reversal of the Neuronal Hyperexcitability and Painful Sensitivity in Prediabetic Rats via Activation of TREK1/2 Channels

TREK channels are crucial for establishing RMP, regulating cellular excitability, and determining background potassium current. Accumulating evidence has demonstrated that the TREK1 channel contributes to slow after hyperpolarization and regulates the frequency of spikes in the retina. Further research also revealed that TREK1 knockout animals have higher spike frequencies of retinal waves.^[^ ^38^ ^]^ Consistent with these previous studies, we found that TREK1 and TREK2 play predominant roles in regulating the excitability of DRG neurons. Moreover, a significant reduction in TREK1/2‐induced K^+^ currents in DRG neurons was observed under the PDNP state. Administration of BL1249 effectively suppressed the hyperexcitability and repetitive firing of these PDNP neurons, resulting in the attenuation of firing frequency, augmentation of the action potential threshold, and amplification of AHP amplitude. Unexpectedly, repeated (tonic) firing neurons responded more strongly to BL1249. However, it is also crucial to identify the molecular characteristics and cell types of tonic‐firing neurons. In future studies, single‐cell RT‐qPCR and single‐cell sequencing should be conducted to further investigate this issue.

On the other hand, to clarify whether TREK1 and TREK2 can be identified as potential molecular targets to alleviate PDNP, BL1249 was intraganglionically injected into PDNP animals. BL1249 effectively reversed the mechanical allodynia and thermal hyperalgesia in PDNP rats. These results are consistent with those of other neuropathic pain animal models, which showed that the activation of TREK1 and TREK2 reverses migraine‐like cutaneous allodynia and SNL‐induced mechanical allodynia in rodents.^[^ ^26^, ^59^ ^]^ However, our study provides novel evidence for alleviating thermal hyperalgesia induced by PDN through activation of TREK1 and TREK2 channels. Therefore, targeting the activation of TREK1 and TREK2 channels could be a potential therapeutic approach for managing chronic pain.

In summary, our findings identify the pivotal role of peripheral Sortilin‐TREK1/2 in nociceptive signaling and contribute to our understanding of the mechanisms underlying PDNP. Furthermore, our study identified a novel therapeutic target for PDNP.

## 4 Conclusion

Collectively, our results present a novel molecular mechanism by which Sortilin negatively regulates the expression of TREK1 and TREK2 channels in DRG neurons, thereby modulating PDNP. These findings indicate that Sortilin overexpression leads to the downregulation of TREK1 and TREK2 channels, which induces neuronal hyperexcitability and pain hypersensitivity. Our study suggests that Sortilin‐TREK1/2 may be a potential therapeutic target for the treatment of PDNP.

## 5 Experimental Section

### Experimental Animals

Experiments were conducted on adult male Sprague–Dawley rats weighing 80–100 g. All experimental protocols were approved by the Animal Use and Protection Committee of the Fourth Military Medical University and were in accordance with the National Institutes of Health Guide for the Care and Use of Laboratory Animals (1996 revision). The methods used to generate an experimental prediabetic model with significant neuropathic pain are previously described.^[^ ^10^ ^]^ The rats were maintained at a controlled temperature (22–23 °C) and light (12:12‐h light–dark cycle). After 1 week of acclimatization, the body weight, FBG concentration, PWMT, and PWTL of the rats were evaluated and the rats were randomly divided into two groups. The rats had access to water ad libitum and were assigned to receive a normal chow or HED (22.5% fat, 27% sugar, 21.5% protein) for 8–12 weeks, ad libitum. Every effort was made to minimize both animal suffering and the number of animals used (*n* = 79 rats with prediabetes and *n* = 108 rats in the control group were included in the study).

### Serum Biochemical Index of Prediabetes

All animals underwent a 12‐h overnight fasting before blood was collected. Blood was collected every 5 days by shearing the tail tips between 8:00 and 9:00 a.m. Blood samples were collected again at the end of the experiments. FBG levels were measured using a one‐touch blood glucose meter (Lifescan Inc., California, USA). The fasting plasma insulin concentration was also measured according to the protocol described by the manufacturer of the 125I‐Insulin Radioimmunoassay Kit (Chemclin Biotech Co., Ltd., Beijing, P. R. China), and then HOMA‐IR was calculated using the following equation: HOMA‐IR = fasting insulin (µIU mL^−1^) × fasting glucose (mmol mL^−1^)/22.5.^[^ ^69^ ^]^

### Intact DRG Preparation and In Vitro Electrophysiological Recordings

Rats were anesthetized with sodium pentobarbital (50 mg kg^−1^, i.p.), and the L4 and L5 DRGs were carefully removed from the vertebral column and placed into an artificial cerebrospinal fluid (ACSF) maintained at 4 °C. After cleaning the connective tissue, the intact ganglia were digested with a mixture of 0.4 mg mL^−1^ trypsin (Sigma‐Aldrich) and 1 mg mL^−1^ type‐A collagenase (Sigma‐Aldrich) for 40 min at 37 °C while being oxygenated by gentle bubbling with 95% O_2_ and 5% CO_2_. After digestion, the intact ganglia were transferred into ACSF and incubated at 28 °C with mixed gas for at least 1 h before being placed into a recording chamber.^[^ ^39^ ^]^ During the recordings, the intact ganglia were kept in position using a small anchor and submerged in a chamber filled with an external solution saturated with mixed gas. The temperature was maintained at 26–28 °C. DRG neurons were visualized using a 40× water‐immersion objective attached to a BX51WI microscope (Olympus, Tokyo, Japan) equipped with infrared differential interference contrast optics. Whole‐cell recording was performed with a HEKA 10.0 amplifier (HEKA, German). Patch pipettes (4–7 MΩ) were pulled from borosilicate glass capillaries using a four‐stage horizontal puller (P97, Sutter Instrument Company, Novato, USA), which gave a series resistance of ≈10 MΩ. All junction potentials were corrected online by adjusting the pipette offset using the HEKA Commander software. Voltage errors were minimized using 80–90% series resistance compensation, and the capacitance artifact was canceled by the patch‐clamp amplifier. The linear leakage currents were digitally subtracted offline. Neurons were selected for further study if they had an RMP more negative than −50 mV and exhibited an overshooting action potential.^[^ ^40^ ^]^ Recordings were obtained from a total of 164 DRG neurons, including 88 cells from control rats (*n* = 31) and 76 cells from HED rats (*n* = 33).

The ACSF contained (in mm): 124 NaCl, 2.5 KCl, 1.2 NaH_2_PO_4_, 1 MgCl_2_, 2 CaCl_2_, 25 NaHCO_3_, and 10 glucose. The internal pipette solution for current‐clamp recording and potassium current measurements contained (in mm): 140 KCl, 2 MgCl_2_, 10 HEPES, and 2 Mg‐ATP (pH 7.4, adjusted by KOH). Osmolarity was adjusted to 290–300 mOsm using sucrose. The bath solution for measuring potassium current contained (in mm): 140 choline‐Cl, 5 KCl, 2 CaCl_2_, 1 MgCl_2_, 10 HEPES, 1 CdCl_2_, 10 glucose, and 1 mm tetrodotoxin (TTX) (pH 7.4, adjusted by KOH, 300 mOsm). Drugs were applied by bath superfusion with a change in the solution in the recording chamber, which was completed within 2 min. Data were acquired with a HEKA 10.0 acquisition system (Molecular Devices) using the FitMaster software. The signals were low‐pass filtered at 2 kHz, sampled at 10 kHz, and analyzed offline. To analyze the active membrane properties in current‐clamp mode, depolarizing current steps of 5 ms duration were applied in 20 pA increments to detect the action potential threshold. Current steps of 500 ms duration and 50 pA increments were used to measure the other kinetics of a single spike, that is, the latency to the first spike and the firing frequency. The AP threshold was determined by differentiating the AP waveform and setting an increasing rate of 10 mV ms^−1^ as the AP inflection point. The AP amplitude was measured from the equipotential point of the threshold to the peak spike. AP duration was measured at the half‐width of the spike. The rise and decay slopes were detected from the AP threshold to the peak.

### Behavioral Testing

1) Mechanical pain sensitivity: All animals were handled extensively and habituated to the test environment for 1 week prior to testing. Mechanical pain sensitivity was assessed in four groups of rats: 1) normal diet versus HED, 2) Spadin‐injected versus vehicle‐injected, 3) siRNA‐sham‐injected versus siRNA‐injected, and 4) AAV‐sham‐injected versus AAV‐injected rats. To quantify the mechanical sensitivity of the hindpaw, the rats were placed in a hanging cage with a metal mesh floor and tested by the manual application of Von Frey filaments to the plantar surface of the hind paw. Each filament of any given force was applied ten times, and the paw withdrawal response frequency (percentage of positive responses to the stimulus) was recorded. The force of filament to elicit a 50% frequency of paw withdrawal was defined as the mechanical threshold.^[^ ^40^ ^]^ 2) Thermal pain sensitivity: to quantify the thermal sensitivity of the hind paws, rats were placed in a clear Plexiglass chamber (15 × 620 × 615 cm) located on an elevated clear glass platform (2 mm thick) and tested with the application of radiant heat to the plantar surface of the hindpaw (Iitc Life Science). The radiant heat source was a high‐intensity projector halogen lamp bulb (100 W) positioned under the glass floor, directly beneath the target area of the hindpaw. The distance between the projector lamp bulb and the lower surface of the glass floor was adjusted to produce a light spot on the floor surface with a diameter of 2–3 mm. The heat stimulus was directed toward the plantar surface of the hind paw of each rat. Five stimuli were repeated at the same site, and the inter‐stimulus interval was 10 min for the same hind paw and 5 min for different hindpaws. Thermal latency was determined automatically by the device, as hindpaw withdrawal resulted in the cut‐off of the heat stimulus. To avoid excessive tissue damage, the heat stimulus was limited to a 50 s duration. The mean thermal latency was calculated by averaging the five measurements.^[^ ^39^ ^]^

### Drugs

Spadin (Tocris, USA), a potential blocker of TRKE1, which is derived from Sortilin, was dissolved in 0.9% sterile saline and percutaneously intraganglionic injected (10 and 20 µm) to the left side DRGs of normal rats to evaluate its effect on pain. BL1249 selectively activated TREK1 and TREK2 channels when the extracellular concentration was at 10 µm.^[^ ^70^ ^]^ The stock solution of BL1249 (1 m) was prepared with DMSO and then diluted to the desired concentration in the patch clamp recording solution on the date the experiment was being performed. All chemicals were purchased from Tocris (USA). Percutaneous intraganglionic administration and validation of small interfering RNA (siRNA) siRNAs, all from GenePharma (China), were performed in siRNA dilution buffer and stored at −20 °C until use. The reliability of the siRNA‐TREK1 in normal rats was tested. The sense and antisense sequences of the siRNAs (A–C) were used. siRNA A: sense 5′‐GGCCAUUAAUGUUAUGAAATT‐3′ and antisense 5′‐UUUCAUAACAUUAAUGGCCTT‐3,′ B: sense 5′‐GUGGAGGACACAUUUAUUATT‐3′ and antisense 5′‐UAAUAAAUGUGUCCUCCACTT‐3,′ C: sense 5′‐GCCGAGUUCAAGGAAACAATT‐3′ and antisense 5′‐UUGUUUC CUUGAACUGGCTT‐3. The reliability of the siRNA targeting TREK2 in normal rats was evaluated. The sense and antisense sequences of siRNA (A–C) were. siRNA A: sense 5′‐CCUGCUGUCAUCUUUAAAUTT‐3′ and antisense 5′‐AUUUAAAGAUGACAGCAGGTT‐3,′ B: sense 5′‐CGCACUUGAUGCUGAUAAUTT‐3′ and antisense 5′‐AUUAUCAGCAUCAAGUGCGTT‐3,′ C: sense 5′‐GAGGCCUGCAAACAGUUAUTT‐3′ and antisense 5′‐AUAACUGUUUGCAGGCCUCTT‐3.′ The ability of the four different Sortilin sequences of siRNAs (A–D) to knockdown Sortilin expression was examined by western blotting and immunofluorescence assays. siRNA A: sense, 5′‐GAAAGUCAGUGGAAAGUAAdTdT‐3′ and antisense 5′‐UUACUUUCCACUGACUUUCdTdT‐3,′ B: sense 5′‐AGUUGGUUGUUAUCCAGAAdTdT‐3′ and antisense 5′‐UUCUGGAUAACAACCAACUdTdT‐3,′ C: sense 5′‐GCUGAUAAGGAUACAACAAdTdT‐3′ and antisense 5′‐UUGUUGUAUCCUUAUCAGCdTdT‐3,′ D: sense 5′‐GUUCAUGCAUGUAGA UGAAdTdT‐3′ and antisense 5′‐UUCAUCUACAUGCAUGAACdTdT‐3.

### Percutaneous Intraganglionic Injection

According to a study by Ferreira et al.,^[^ ^71^ ^]^ the injecting needle was prepared by inverting the position of the gingival needle (30G) relative to its plastic syringe connector, which was tightly connected to the metal piece. After anesthetization with isoflurane, the rats were shaved on their lower backs and placed over a small cylinder to elevate the lumbar region. The puncture point was defined at 1.5 cm laterally to the vertebral column, ≈0.5 cm caudal from a virtual line passing over the rostral borders of the iliac crests. To facilitate skin puncture, a larger needle (19G) was used to penetrate the skin of the rat at the puncture point. Subsequently, the injection needle was inserted through the muscle toward the intervertebral space (L4 and L5 or L5 and L6) until the tip touched the lateral region of the vertebrae. To reach the space between the transverse processes of the vertebrae, delicate movements of the needle were made until bone resistance diminished and a paw flinch reflex was observed. The paw flinch reflex indicated that the needle tip had penetrated the DRG. 50 µm siRNA TREK1 or 50 µm siRNA TREK2 were then injected into the DRG at levels of L4, and L5, continuously for 3 days. For drug delivery, the rats were held gently and a 10 µL volume of either vehicle (0.9% saline) or siRNA (10 µL, 50 nm) was infused, followed by a flush of 10 µL sterile saline.

### Intra DRG Injection

The rats were anesthetized with sodium pentobarbital (50 mg kg^−1^, i.p.), and the fur was shaved over the lower back. The vertebral plate was exposed at L2‐S1. The left L4‐5 DRGs were exposed via partial laminectomy. A volume of 1 µL of AAV (rAAV‐hSyn‐Sort1‐P2A‐EGFP‐WPREs, BrainVTA Co., Ltd., China, 5.60 × 10^12^ vg mL^−1^) was injected into each DRG using a Hamilton syringe attached to a 35 ga. Scrambled vector controls received the same volume of AAV‐GFP and underwent hemilaminectomy. Following the injections, the surrounding muscles and overlying skin of the incision were sewn up. The injection location was confirmed after the animals were euthanized. Behavioral tests were conducted 4 weeks postoperatively. Animals that exhibited walking abnormalities were excluded from behavioral tests.^[^ ^72^ ^]^

### Immunofluorescence

Rats were anesthetized and transcardially perfused with saline, followed by 4% paraformaldehyde. DRGs from the lumbar regions of the spinal column were removed, post‐fixed overnight in 4% paraformaldehyde, and cryoprotected in 30% sucrose at 4 °C until the tissue sank to the bottom of the container. Transverse DRG sections (14 µm) were cut on a DRG. Double immunofluorescence labeling was used to visualize TREK1, TREK2 channels, and Sortilin. Briefly, the sections were incubated in a solution containing 0.3% Triton X‐100 and 1% bovine serum albumin for 3 h at room temperature. The sections were then incubated with anti‐TREK (TREK 1 and TREK 2, rabbit, 1:200; Alomone Labs) channel antibodies, monoclonal anti‐NF200 (mouse, 1:200, Sigma‐Aldrich) antibody, monoclonal anti‐CGRP (mouse, 1:100, Millipore, and rabbit, 1:200, Sigma‐Aldrich) antibody, and anti‐Sortilin antibody (rabbit, 1:200, Alomone Labs, and sheep, 1:300, Abcam) for 24 h at 4 °C. After three washes with phosphate‐buffered saline, the sections were incubated with secondary antibodies Cy3 (goat anti‐rabbit IgG, Sigma‐Aldrich) and FITC (goat anti‐mouse IgG and goat anti‐sheep IgG, Sigma‐Aldrich) for 3 h at room temperature. All images were captured using an Olympus confocal microscope and processed using ImageJ software.

### Western Blot

DRGs were collected and homogenized in ice‐cold lysis buffer containing (in mm): 50 Tris‐HCl, pH 7.4, 150 NaCl, 5 EDTA, 1% Triton X‐100, 0.5% sodium deoxycholate, 0.1% sodium dodecyl sulfate, and standard protease inhibitors. The insoluble material was removed by centrifugation (13000 rpm, 10 min), and the supernatant was collected. The protein concentration of each sample was determined using the bicinchoninic acid method with a MICRO BCA protein assay kit (Pierce). The membrane blots were blocked with 10% non‐fat dry milk for 12 h and incubated with primary antibodies: anti‐TREK1 (1:100), anti‐TREK2 (1:200), and anti‐Sortilin (1:200) overnight at 4 °C. The membranes were then incubated with a horseradish peroxidase‐conjugated goat anti‐rabbit secondary antibody (1:10 000, Amersham Biosciences) for 2 h at room temperature. To normalize the loaded samples, a mouse monoclonal anti‐tubulin antibody (1:5000; GE Healthcare) was used, followed by incubation with horseradish peroxidase‐conjugated goat anti‐rabbit IgG (1:5000; Pierce). Membranes were incubated with enhanced chemiluminescence reagents (Pierce), and images of the membranes were acquired using the CHEMIL‐MAGER chemiluminescence imaging system and analyzed using ImageJ software. The densities of the bands of interest (TREK1, TREK2, and Sortilin) were measured and normalized to that of the tubulin band (Amersham Biosciences).

### Co‐Immunoprecipitation Assay

The interaction between Sortilin, TREK1, and TREK2 was investigated using a co‐precipitation assay with the Pierce Classic IP Kit (Cat. No. 26146, Thermo, USA). Briefly, DRGs were collected and homogenized in an ice‐cold lysis buffer provided by the manufacturer. Insoluble material was removed by centrifugation (13 000 rpm for 10 min at 4 °C), and the supernatant was collected. Following this, the supernatants were incubated with the Sortilin antibody at 4 °C overnight to form the immune complex. The immune complex was subjected to western blot analysis as described above. The interaction between Sortilin and TREK1/2 in each sample was determined using western blot analysis.

### Real‐Time Quantitative PCR

Total RNA from DRG tissues was extracted using TRIzol reagent (Thermo Fisher Scientific, 15596026CN). Subsequently, cDNA synthesis was performed on 1 µg of total RNA utilizing the Transcriptor First Strand cDNA Synthesis Kit (Roche, Switzerland, 04896866001). Quantitative RT‐PCR was performed with Power SYBR Green PCR Master Mix (4367659; Thermo Fisher Scientific) using a CFX96TM Real‐Time system (Bio‐Rad). The PCR cycling conditions were designed as follows: 10 min at 95 °C for initial denaturation, 40 cycles of 10 s at 95 °C for denaturation, 15 s at 60 °C for annealing, and 20 s at 72 °C for extension. The primer sequences were shown as follows: Kcnk2:5′‐AAggAAgAggTgggAgAgTT‐3′ and 5′‐CACgCTggAACTTgTCATAgA‐3′; Kcnk10:5′‐CTgTTCCTCgA CTCTCCATTTC‐3′ and 5′‐CCACgACCACCACAAAgAT‐3′; Actin: 5′‐AgATCCTgACCgAgCgTggC‐3′ and 5′‐CCAgggAggAAgAggATgCg‐3. The relative amount of mRNA was quantified to the internal β‐actin control, and normalized to the corresponding control.

### Statistical Analysis

All results are presented as the mean ± SEM. No parametric *U* test, *t*‐test, or analysis of variance (ANOVA) for random measures, followed by a post‐hoc Bonferroni test, were used to determine statistically significant differences. The data collected over time or recorded with continuous stimuli among the groups were compared using a two‐way repeated‐measures ANOVA. Statistical significance was set at *p* < 0.05. ImageJ, GraphPad, OriginPro 8.5.1, and SPSS 25.0, were used for data statistics and analysis (see Table S3, Supporting Information, for further details).

## Conflict of Interest

The authors declare no conflict of interest.

## Author Contributions

W.S., F.Y., Y.W., and Y.Y. contributed equally to this work. They performed the experiments and analyzed the data. W.S. and J.‐J.W. drafted the manuscript. R.D. and Z.‐X.L. were partially involved in conducting behavioral assays. X.‐L.W. assisted in the preparation of animal models. J.‐J.W. and J.C. are the corresponding authors. They designed the research project, supervised experiments and critically reviewed the manuscript.

## Supporting information

Supporting Information

## Data Availability

The data that support the findings of this study are available from the corresponding author upon reasonable request.

## References

[1] L. A. Zilliox , Clin. Geriatr. Med. 2021, 37, 253. 33858608 10.1016/j.cger.2020.12.001

[2] R. Khan , Z. Chua , J. C. Tan , Y. Yang , Z. Liao , Y. Zhao , Medicina 2019, 55, 546. 31470636 10.3390/medicina55090546 PMC6780236

[3] D. Ziegler , N. Papanas , A. I. Vinik , J. E. Shaw , Handb. Clin. Neurol. 2014, 126, 3. 25410210 10.1016/B978-0-444-53480-4.00001-1

[4] N. Papanas , D. Ziegler , Curr. Diabetes Rep. 2012, 12, 376. 10.1007/s11892-012-0278-3 22562652

[5] J. R. Singleton , A. G. Smith , J. Russell , E. L. Feldman , Curr. Treat. Options Neurol. 2005, 7, 33. 15610705 10.1007/s11940-005-0004-4

[6] M. J. Hossain , M. D. Kendig , B. M. Wild , T. Issar , A. V. Krishnan , M. J. Morris , R. Arnold , Biomedicines 2020, 8, 313. 32872256 10.3390/biomedicines8090313 PMC7555926

[7] S. A. Eid , E. L. Feldman , Dis. Models Mech. 2021, 14, dmm049337. 10.1242/dmm.049337 PMC8592018 34762126

[8] J. Jende , Z. Kender , C. Mooshage , J. B. Groener , L. Alvarez‐Ramos , J. Kollmer , A. Juerchott , A. Hahn , S. Heiland , P. Nawroth , M. Bendszus , S. Kopf , F. T. Kurz , Front. Neurosci. 2021, 15, 642589. 33746707 10.3389/fnins.2021.642589 PMC7966816

[9] A. Bischoff , Z. Neurol. 1973, 205, 257. 4130588 10.1007/BF00316020

[10] F. Xie , H. Fu , J. F. Hou , K. Jiao , M. Costigan , J. Chen , PLoS One 2013, 8, e57427. 23451227 10.1371/journal.pone.0057427 PMC3581455

[11] M. Sajic , A. E. Rumora , A. A. Kanhai , G. Dentoni , S. Varatharajah , C. Casey , R. Brown , F. Peters , L. M. Hinder , M. G. Savelieff , E. L. Feldman , K. J. Smith , J. Neurosci. 2021, 41, 4321. 33785643 10.1523/JNEUROSCI.1852-20.2021 PMC8143198

[12] A. E. Rumora , K. Guo , L. M. Hinder , P. D. O'Brien , J. M. Hayes , J. Hur , E. L. Feldman , Front. Physiol. 2022, 13, 921942. 36072849 10.3389/fphys.2022.921942 PMC9441493

[13] A. E. Rumora , G. Lograsso , J. M. Hayes , F. E. Mendelson , M. A. Tabbey , J. A. Haidar , S. I. Lentz , E. L. Feldman , J. Neurosci. 2019, 39, 3770. 30886017 10.1523/JNEUROSCI.3173-18.2019 PMC6510336

[14] O. Pongs , in Inhibitory Regulation of Excitatory Neurotransmission, Springer, Berlin 2008, pp. 145–161.

[15] J. Wang , S. W. Ou , Y. J. Wang , Channels 2017, 11, 534.28922053

[16] J. Busserolles , C. Tsantoulas , A. Eschalier , G. J. Lopez , Pain 2016, 157, S7. 26785158 10.1097/j.pain.0000000000000368

[17] V. Viatchenko‐Karpinski , J. Ling , J. G. Gu , Mol. Brain 2018, 11, 40. 29980241 10.1186/s13041-018-0384-5 PMC6035395

[18] J. A. Lamas , L. Rueda‐Ruzafa , S. Herrera‐Perez , Int. J. Mol. Sci. 2019, 20, 2371. 31091651 10.3390/ijms20102371 PMC6566417

[19] M. Lolicato , P. M. Riegelhaupt , C. Arrigoni , K. A. Clark , D. J. Minor , Neuron 2014, 84, 1198. 25500157 10.1016/j.neuron.2014.11.017 PMC4270892

[20] X. Y. Li , H. Toyoda , Brain Res. Bull. 2015, 119, 73. 26321392 10.1016/j.brainresbull.2015.08.007

[21] A. Djillani , J. Mazella , C. Heurteaux , M. Borsotto , Front. Pharmacol. 2019, 10, 379. 31031627 10.3389/fphar.2019.00379 PMC6470294

[22] D. Kang , D. Kim , Am. J. Physiol. Cell Physiol. 2006, 291, C138. 16495368 10.1152/ajpcell.00629.2005

[23] E. Honore , Nat. Rev. Neurosci. 2007, 8, 251. 17375039 10.1038/nrn2117

[24] V. Pereira , J. Busserolles , M. Christin , M. Devilliers , L. Poupon , W. Legha , A. Alloui , Y. Aissouni , E. Bourinet , F. Lesage , A. Eschalier , M. Lazdunski , J. Noel , Pain 2014, 155, 2534. 25239074 10.1016/j.pain.2014.09.013

[25] P. Royal , A. Andres‐Bilbe , P. P. Avalos , C. Verkest , B. Wdziekonski , S. Schaub , Baron A. , F. Lesage , X. Gasull , J. Levitz , G. Sandoz , Neuron 2019, 101, 232. 30573346 10.1016/j.neuron.2018.11.039

[26] G. Garcia , K. A. Mendez‐Resendiz , N. Oviedo , J. Murbartian , J. Neurochem. 2021, 157, 2039. 33006141 10.1111/jnc.15204

[27] G. Garcia , V. A. Martinez‐Rojas , J. Murbartian , Behav. Brain Res. 2021, 413, 113446. 34224765 10.1016/j.bbr.2021.113446

[28] E. S. Schwartz , A. Xie , La J. H. , G. F. Gebhart , Pain 2015, 156, 1537. 25915147 10.1097/j.pain.0000000000000201 PMC5260933

[29] J. Descoeur , V. Pereira , A. Pizzoccaro , A. Francois , B. Ling , V. Maffre , B. Couette , J. Busserolles , C. Courteix , J. Noel , M. Lazdunski , A. Eschalier , N. Authier , E. Bourinet , EMBO Mol. Med. 2011, 3, 266. 21438154 10.1002/emmm.201100134 PMC3377073

[30] G. Sandoz , S. Thummler , F. Duprat , S. Feliciangeli , J. Vinh , P. Escoubas , N. Guy , M. Lazdunski , F. Lesage , EMBO J. 2006, 25, 5864. 17110924 10.1038/sj.emboj.7601437 PMC1698884

[31] G. Sandoz , M. P. Tardy , S. Thummler , S. Feliciangeli , M. Lazdunski , F. Lesage , J. Neurosci. 2008, 28, 8545. 18716213 10.1523/JNEUROSCI.1962-08.2008 PMC6671063

[32] E. Kim , E. M. Hwang , O. Yarishkin , J. C. Yoo , D. Kim , N. Park , M. Cho , Y. S. Lee , C. H. Sun , G. S. Yi , J. Yoo , D. Kang , J. Han , S. G. Hong , J. Y. Park , Biochem. Biophys. Res. Commun. 2010, 395, 244. 20362547 10.1016/j.bbrc.2010.03.171

[33] X. Su , L. Chen , X. Chen , C. Dai , B. Wang , Bosnian J. Basic Med. Sci. 2022, 22, 340. 10.17305/bjbms.2021.6601 PMC9162750 34784266

[34] J. Mazella , O. Petrault , G. Lucas , E. Deval , S. Beraud‐Dufour , C. Gandin , M. El‐Yacoubi , C. Widmann , A. Guyon , E. Chevet , S. Taouji , G. Conductier , A. Corinus , T. Coppola , G. Gobbi , J. L. Nahon , C. Heurteaux , M. Borsotto , PLoS Biol. 2010, 8, e1000355. 20405001 10.1371/journal.pbio.1000355 PMC2854129

[35] S. J. Korn , J. G. Trapani , IEEE Trans. Nanobiosci. 2005, 4, 21. 10.1109/tnb.2004.842466 15816169

[36] A. Mathie , E. L. Veale , Pfluegers Arch. 2015, 467, 931. 25420526 10.1007/s00424-014-1655-3

[37] A. J. Patel , E. Honore , Trends Neurosci. 2001, 24, 339. 11356506 10.1016/s0166-2236(00)01810-5

[38] K. J. Ford , D. A. Arroyo , J. N. Kay , E. E. Lloyd , R. J. Bryan , J. R. Sanes , M. B. Feller , J. Neurophysiol. 2013, 109, 2250. 23390312 10.1152/jn.01085.2012 PMC3652223

[39] W. Sun , B. Miao , X. C. Wang , J. H. Duan , W. T. Wang , F. Kuang , R. G. Xie , J. L. Xing , H. Xu , X. J. Song , C. Luo , S. J. Hu , Brain 2012, 135, 359. 22271663 10.1093/brain/awr345

[40] W. Sun , F. Yang , Y. Wang , H. Fu , Y. Yang , C. L. Li , X. L. Wang , Q. Lin , J. Chen , Neuropharmacology 2017, 113, 217. 27743933 10.1016/j.neuropharm.2016.10.012

[41] L. Li , Y. Liu , W. Hu , J. Yang , S. Ma , Z. Tian , Z. Cao , K. Pan , M. Jiang , X. Liu , S. Wu , C. Luo , R. G. Xie , Front. Mol. Neurosci. 2023, 16, 1144614. 37860084 10.3389/fnmol.2023.1144614 PMC10582564

[42] J. Pan , Y. Zhao , R. Sang , R. Yang , J. Bao , Y. Wu , Y. Fei , J. Wu , G. Chen , Pain 2023, 164, e286. 36508175 10.1097/j.pain.0000000000002837

[43] L. Catacuzzeno , B. Fioretti , D. Pietrobon , F. Franciolini , J. Physiol. 2008, 586, 5101. 18772201 10.1113/jphysiol.2008.159384 PMC2652152

[44] E. K. Yang , K. Takimoto , Y. Hayashi , W. C. de Groat , N. Yoshimura , Neuroscience 2004, 123, 867. 14751280 10.1016/j.neuroscience.2003.11.014

[45] B. Wang , L. Ma , X. Guo , Du S. , X. Feng , Y. Liang , G. Govindarajalu , S. Wu , T. Liu , H. Li , S. Patel , A. Bekker , H. Hu , Y. X. Tao , Brain 2023, 146, 3866. 37012681 10.1093/brain/awad110 PMC10473565

[46] S. R. Chen , Y. Q. Cai , H. L. Pan , J. Neurochem. 2009, 110, 352. 19457113 10.1111/j.1471-4159.2009.06138.x PMC2905800

[47] C. D. Sarantopoulos , J. B. Mccallum , M. Rigaud , A. Fuchs , W. M. Kwok , Q. H. Hogan , Brain Res. 2007, 1132, 84. 17184741 10.1016/j.brainres.2006.11.055 PMC2692681

[48] R. Lu , R. Lukowski , M. Sausbier , D. D. Zhang , M. Sisignano , C. D. Schuh , R. Kuner , P. Ruth , G. Geisslinger , A. Schmidtko , Pain 2014, 155, 556. 24333777 10.1016/j.pain.2013.12.005

[49] F. X. Zhang , V. M. Gadotti , I. A. Souza , L. Chen , G. W. Zamponi , Cell Rep. 2018, 22, 1956. 29466724 10.1016/j.celrep.2018.01.073

[50] C. Acosta , L. Djouhri , R. Watkins , C. Berry , K. Bromage , S. N. Lawson , J. Neurosci. 2014, 34, 1494. 24453337 10.1523/JNEUROSCI.4528-13.2014 PMC3898302

[51] E. A. Konnova , A. F. Deftu , S. C. P. Chu , G. Kirschmann , I. Decosterd , M. R. Suter , Glia 2024, 72, 677. 38108588 10.1002/glia.24496

[52] M. Kwon , I. Y. Jung , M. Cha , B. H. Lee , Front. Pharmacol. 2021, 12, 759730. 34955831 10.3389/fphar.2021.759730 PMC8694709

[53] W. Li , S. B. Gao , C. X. Lv , Y. Wu , Z. H. Guo , J. P. Ding , T. Xu , J. Cell. Physiol. 2007, 212, 348. 17523149 10.1002/jcp.21007

[54] X. Ma , L. S. Miraucourt , H. Qiu , R. Sharif‐Naeini , A. Khadra , J. Neurosci. 2023, 43, 5608. 37451982 10.1523/JNEUROSCI.0426-23.2023 PMC10401647

[55] S. Blin , I. B. Soussia , E. Kim , F. Brau , D. Kang , F. Lesage , D. Bichet , Proc. Natl. Acad. Sci. USA 2016, 113, 4200. 27035965 10.1073/pnas.1522748113 PMC4839434

[56] J. R. Schwarz , J. Physiol. 2021, 599, 4427. 34425634 10.1113/JP281723

[57] H. Kanda , J. Ling , S. Tonomura , K. Noguchi , S. Matalon , J. G. Gu , Neuron 2019, 104, 960. 31630908 10.1016/j.neuron.2019.08.042 PMC6895425

[58] J. Noel , K. Zimmermann , J. Busserolles , E. Deval , A. Alloui , S. Diochot , N. Guy , M. Borsotto , P. Reeh , A. Eschalier , M. Lazdunski , EMBO J. 2009, 28, 1308. 19279663 10.1038/emboj.2009.57 PMC2683043

[59] A. Alloui , K. Zimmermann , J. Mamet , F. Duprat , J. Noel , J. Chemin , N. Guy , N. Blondeau , N. Voilley , C. Rubat‐Coudert , M. Borsotto , G. Romey , C. Heurteaux , P. Reeh , A. Eschalier , M. Lazdunski , EMBO J. 2006, 25, 2368. 16675954 10.1038/sj.emboj.7601116 PMC1478167

[60] F. Lesage , C. Terrenoire , G. Romey , M. Lazdunski , J. Biol. Chem. 2000, 275, 28398. 10880510 10.1074/jbc.M002822200

[61] P. P. Avalos , A. A. Chassot , A. Landra‐Willm , G. Sandoz , Neurosci. Lett. 2022, 773, 136494. 35114333 10.1016/j.neulet.2022.136494

[62] P. Rivas‐Ramirez , A. Reboreda , L. Rueda‐Ruzafa , S. Herrera‐Perez , J. A. Lamas , Int. J. Mol. Sci. 2020, 21, 389. 31936257 10.3390/ijms21020389 PMC7014146

[63] J. Noel , G. Sandoz , F. Lesage , Channels 2011, 5, 402. 21829087 10.4161/chan.5.5.16469 PMC3265763

[64] A. Salasova , G. Monti , O. M. Andersen , A. Nykjaer , Mol. Neurodegener. 2022, 17, 74. 36397124 10.1186/s13024-022-00576-2 PMC9673319

[65] N. M. El‐Khodary , H. Dabees , R. H. Werida , Nutr. Diabetes 2022, 12, 33. 35732620 10.1038/s41387-022-00210-6 PMC9217798

[66] J. Li , D. J. Matye , T. Li , J. Biol. Chem. 2015, 290, 11526. 25805502 10.1074/jbc.M115.641225 PMC4416856

[67] M. Richner , L. T. Pallesen , M. Ulrichsen , E. T. Poulsen , T. H. Holm , H. Login , A. Castonguay , L. E. Lorenzo , N. P. Goncalves , O. M. Andersen , K. Lykke‐Hartmann , J. J. Enghild , L. Ronn , I. J. Malik , Y. De Koninck , O. J. Bjerrum , C. B. Vaegter , A. Nykjaer , Sci. Adv. 2019, 5, eaav9946. 31223654 10.1126/sciadv.aav9946 PMC6584543

[68] I. Ho , X. Liu , Y. Zou , T. Liu , W. Hu , H. Chan , Y. Tian , Y. Zhang , Q. Li , S. Kou , C. S. Chan , T. Gin , C. Cheng , S. H. Wong , J. Yu , L. Zhang , W. Wu , M. Chan , Theranostics 2019, 9, 1651. 31037129 10.7150/thno.29703 PMC6485195

[69] E. M. Seliem , M. E. Azab , R. S. Ismail , A. A. Nafeaa , B. S. Alotaibi , W. A. Negm , Life 2022, 12, 693. 35629362 10.3390/life12050693 PMC9144088

[70] L. Pope , C. Arrigoni , H. Lou , C. Bryant , A. Gallardo‐Godoy , A. R. Renslo , D. J. Minor , ACS Chem. Neurosci. 2018, 9, 3153. 30089357 10.1021/acschemneuro.8b00337 PMC6302903

[71] L. F. Ferrari , F. Q. Cunha , C. A. Parada , S. H. Ferreira , J. Neurosci. Methods 2007, 159, 236. 16973217 10.1016/j.jneumeth.2006.07.025

[72] X. Yuan , S. Han , F. Zhao , A. Manyande , F. Gao , J. Wang , W. Zhang , X. Tian , Front. Neurol. 2023, 14, 1138933. 37114234 10.3389/fneur.2023.1138933 PMC10126363

