# Supplementary material for: Sortilin‐Mediated Inhibition of TREK1/2 Channels in Primary Sensory Neurons Promotes Prediabetic Neuropathic Pain
Source: Adv Sci (Weinh). 2024 Apr 16;11(23):2310295. doi: 10.1002/advs.202310295 (PMC11187941; doi:10.1002/advs.202310295)
Supplement: Supplementary file 1 — Supporting Information [file ADVS-11-2310295-s001.docx]

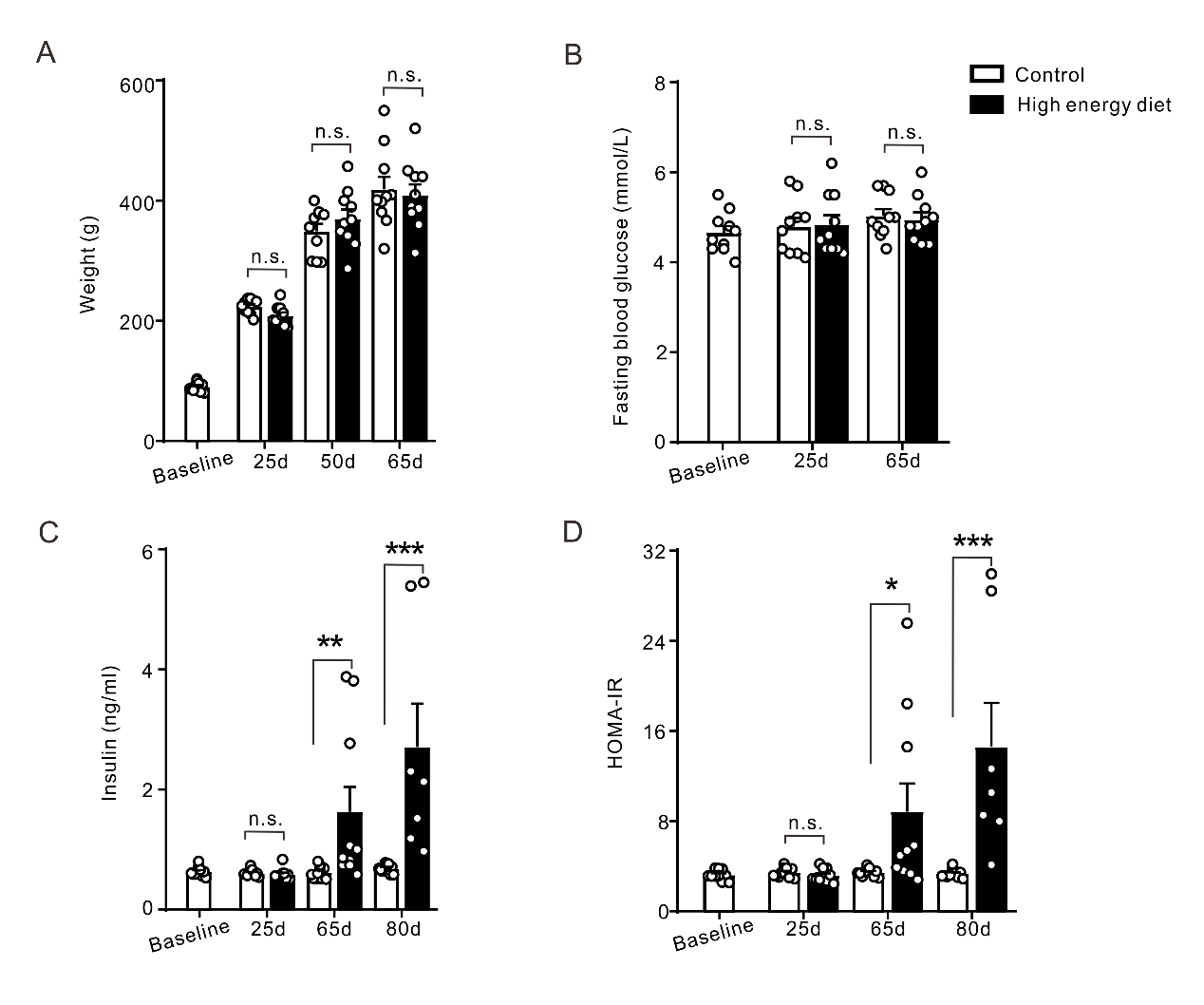


**Fig.S1** The effects of HED on blood biochemistry and HOMA-IR assays with time courses. (A) The histogram shows that the body weight of rats was increased both in HED group and normal chow group. n.s., no significance, Independent Samples *t*-test, n = 10 rats per group. (B) Up to 65 days on HED, the fasting blood glucose of rats did not significantly elevate. n.s., no significance, Independent Samples *t*-test, n = 10 rats per group. (C) This panel indicates that the fasting plasma insulin level of rats was remarkably upregulated at the 65^th^ d and the 80^th^ d after HED compared with control rats, respectively. n.s., no significance, ** *P* < 0.01, *** *P* < 0.001, Mann Whitney U test, n = 7-10 rats per group. (D) The HOMA-IR of rats was elevated by HED. n.s., no significance, Independent Samples *t*-test, * *P* < 0.05, *** *P* < 0.001, Mann Whitney U test, n = 7-10 rats per group.


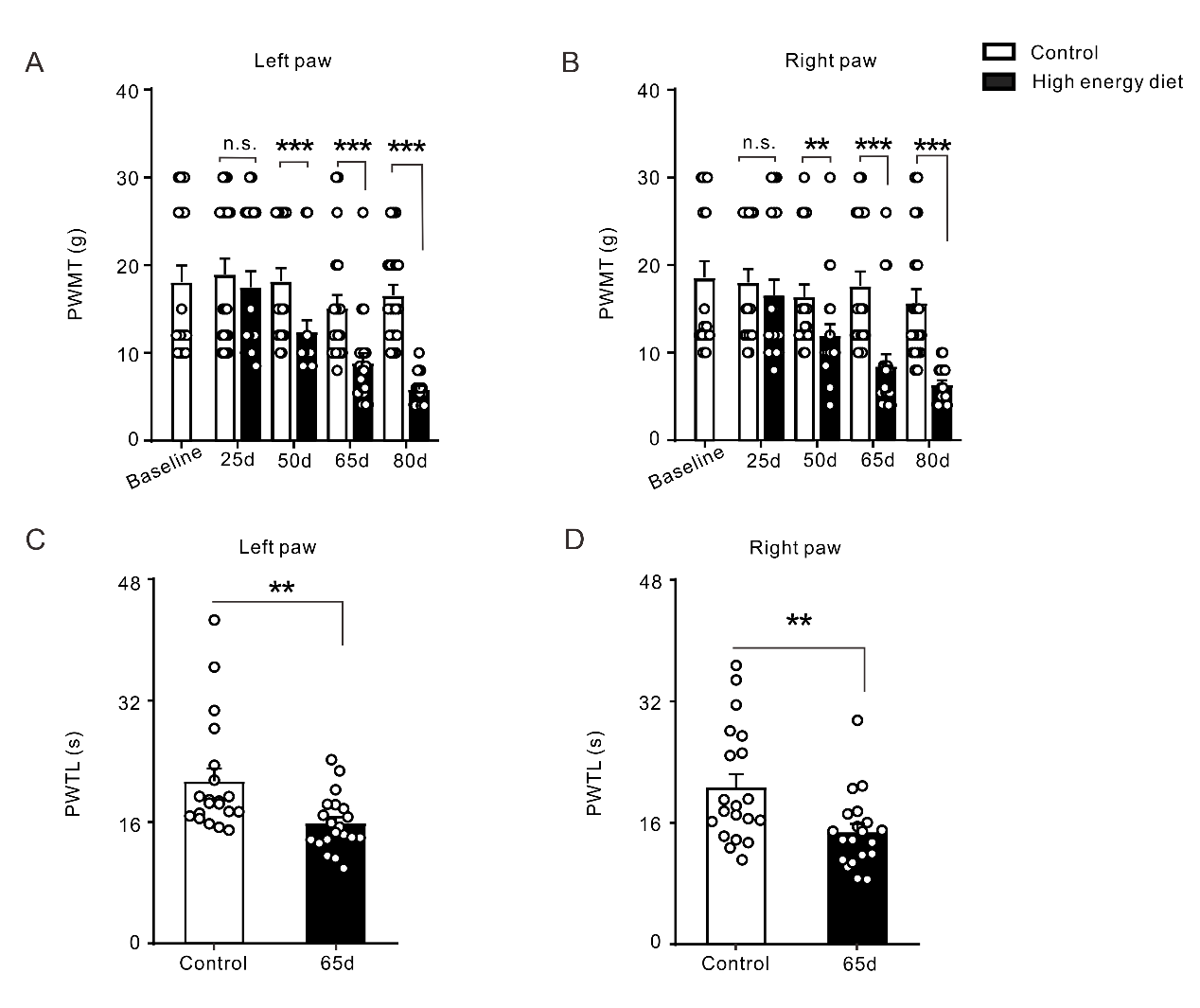


**Fig.S2** Time courses of the effects of HED on pain behavioral assays. (A, B) Mechanical allodynia of bilateral hindpaws was developed in rats with HED. Note that the value of PWMT was reduced on the 50^th^ day following high-energy diet. n.s., no significance, ** *P* < 0.01, *** *P* < 0.001, Mann Whitney U test, n = 17-20 rats per group. (C, D) Thermal hyperalgesia of bilateral hindpaws was developed in rats feeding HED. The value of PWTL in bilateral hindpaws was dramatically decreased on the 65^th^ day after HED compared to controls. ** *P* < 0.01, Mann Whitney U test, n = 20 rats per group.


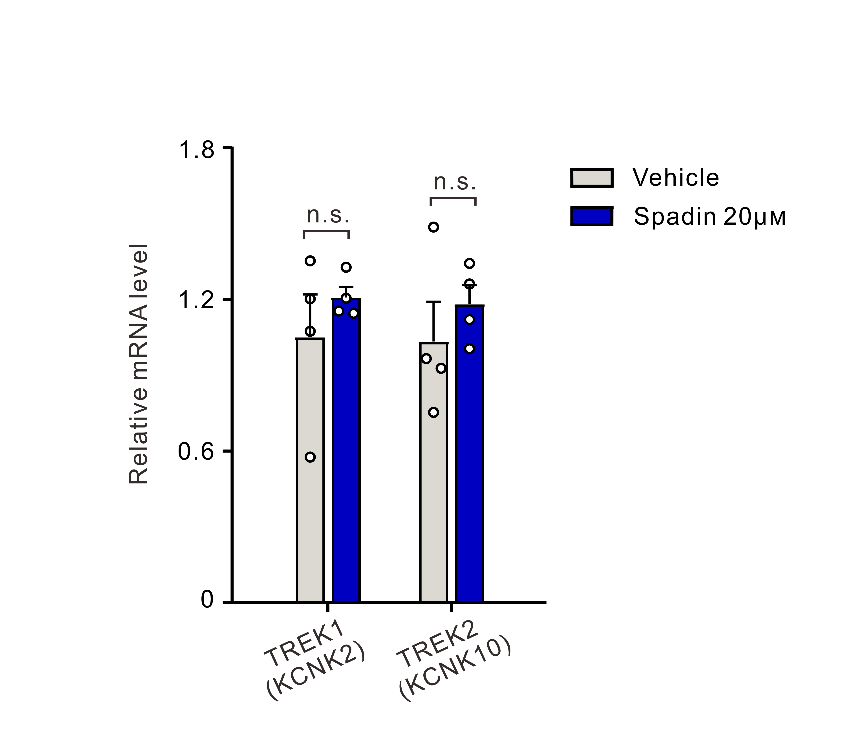


**Fig.S3** The mRNA expressions of KCNK2 and KCNK10 in DRGs unchanged after vehicle and Spadin administration. The mRNA expressions of KCNK2 and KCNK10 was measured by RT-qPCR. The quantitative data indicate that the mRNA expression of KCNK2 and KCNK10 was not affected by Spadin injection. n.s., no significance, Independent Samples *t*-test, n = 4 rats per group.


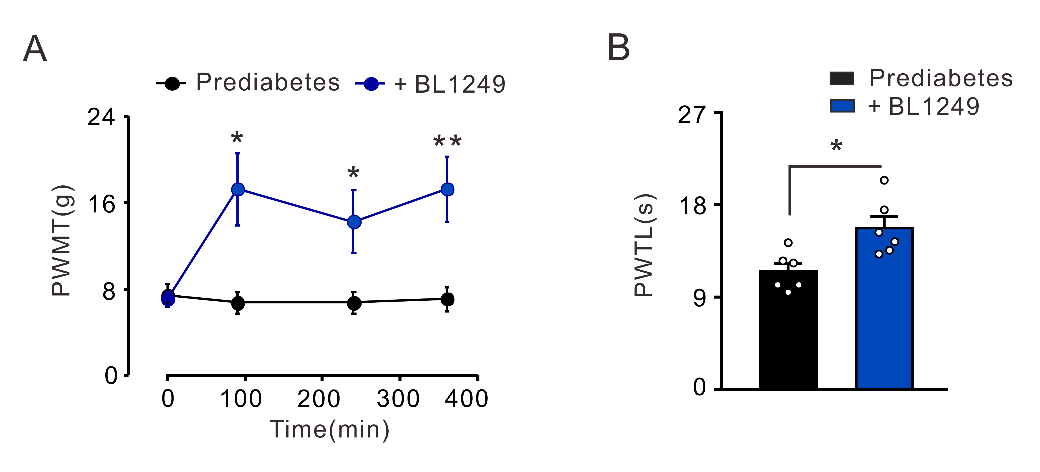


**Fig.S4** Percutaneous intraganglia injection of BL1249 attenuates pain hypersensitization in prediabetic rats. (A) The time course curves show that the PWMT of the ipsilateral hinderpaws significantly increased, starting from 90 min after 10 μᴍ BL1249 injected. * *P* < 0.05, ** *P* < 0.01, Two-way ANOVA RM, n = 6 rats per group. (B) The histogram indicates that the administration of 10 μᴍ BL1249 resulted in a significant increase in the PWTL of rats at the 90 min mark. * *P* < 0.05, Independent Samples *t*-test, n = 6 rats per group.


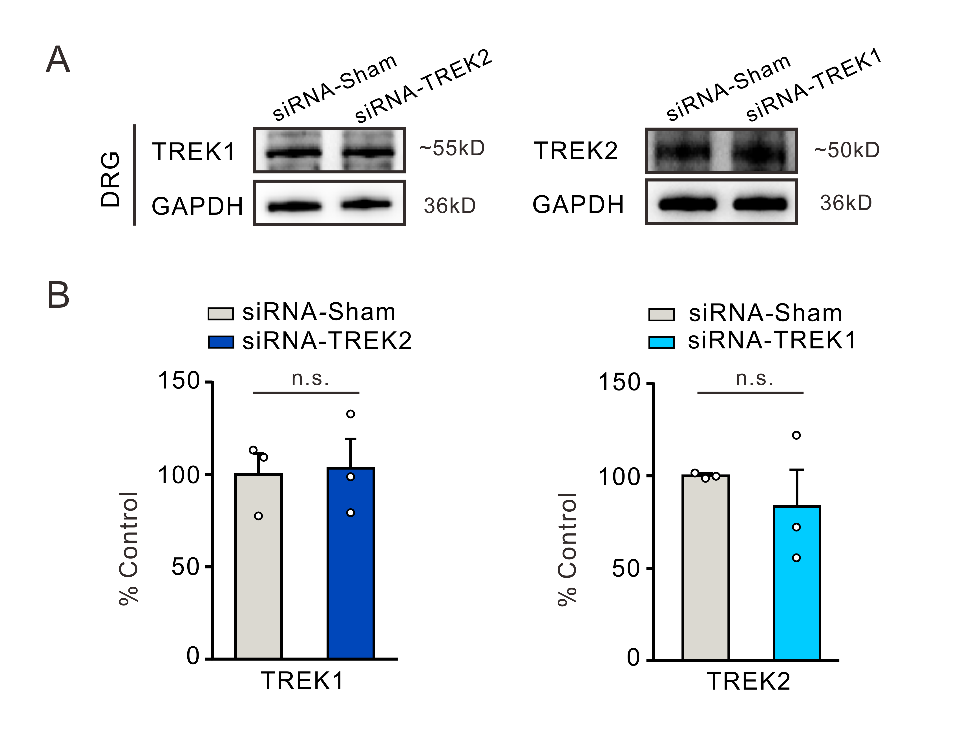


**Fig.S5** Targeted TREK1 siRNA injection suppresses the expression of TREK1 but not TREK2, and vice versa. (A) Left panel: representative bands of TREK1 in ipsilateral L4-5 DRG neurons after siRNA-TREK2 injection. Right panel: representative bands of TREK2 in ipsilateral L4-5 DRG neurons after siRNA-TREK1 injection. (B) The quantitative data of immunoreactive bands indicate that the expression of TREK1 was not affected by siRNA-TREK2 injection, and vice versa. n.s., no significance, Independent Samples *t*-test (left pannel) and Mann Whitney U test (right pannel), n = 3 rats per group.

**Table S1** Key resources of reagents and software used in the current study.

Table S1 Key resources of reagents and software used in the current study

| **Reagent or resource** | **Source** | **Identifier** |
| --- | --- | --- |
| **Antibody** | |  |
| Anti rabbit Sortilin antibody | Alomone, [Israel](javascript:;) | Cat# ANT-009 |
| Anti rabbit TREK-1 antibody | Alomone, [Israel](javascript:;) | Cat# APC-047 |
| Anti rabbit TREK-2 antibody | Alomone, [Israel](javascript:;) | Cat# APC-055 |
| Anti sheep Sortilin antibody | Abcam, UK | Cat# ab72019 |
| Anti goat CGRP antibody | Abcam, UK | Cat# ab36001 |
| Anti mouse NF200 antibody | Sigma-Aldrich, USA | Cat# N0142 |
| Anti chicken GFP antibody | Abcam | Cat# ab13970 |
| IB4-FITC | Sigma-Aldrich, USA | Cat# L2895 |
| Anti rabbit GAPDH antibody | Cell Signaling, USA | Cat# 5174 |
| Anti β-actin antibody | Sigma-Aldrich, USA | Cat# A1978 |
| Normal Rabbit IgG | Sigma-Aldrich, USA | Cat# NI01 |
| Anti mouse IgG FITC antibody | Sigma-Aldrich, USA | Cat# F9137 |
| Anti goat IgG FITC antibody | Jackson immunoResearch | Cat# 705-095-147 |
| Anti-chicken IgY (IgG) antibody | Jackson immunoResearch | Cat# 703-545-155 |
| Anti-rabbit IgG Cy3 antibody | Jackson immunoResearch | Cat# 711-165-152 |
| Anti rabbit IgG Cy3 antibody | Sigma-Aldrich, USA | Cat# C2306 |
| Anti sheep IgG FITC antibody | Sigma-Aldrich, USA | Cat# SAB3700722 |
| Anti rabbit IgG HRP antibody | ZSGB-BIO, China | Cat# ZB2301 |
| Anti mouse IgG HRP antibody | ZSGB-BIO, China | Cat# ZB2305 |
| **Drugs** |  |  |
| BL-1249 | Tocris, USA | Cat# 3797 |
| Spadin | Sigma-Aldrich, USA | Cat# F9634 |
| **Critical Commercial Assays** | |  |
| [BCA Protein Assay Kit](https://www.thermofisher.com/order/catalog/product/A53225) | Thermo Fisher, USA | Cat# A53225 |
| Pierce Classic IP Kit | Thermo Fisher, USA | Cat# 26146 |
| Clean-Blot™ IP Kit | Thermo Fisher, USA | Cat# 21230 |
| TRIzolTM reagent | Thermo Fisher, USA | Cat# 15596026CN |
| Transcriptor First Strand cDNA Synthesis Kit | Roche Life Science Products | Cat# 04896866001 |
| Power SYBR Green PCR Master Mix | Thermo Fisher, USA | Cat# 4367659 |
| **Experimental Models: Organisms/Strains** | |  |
| Rat: (Sprague Dawley, SD) | Laboratory Animal Center of the Fourth Military Medical University | N/A |
| **Software and Algorithms** | |  |
| Statistical software | <https://www.ibm.com/nl-en/products/>spss-modeler/ | SPSS 25.0 |
| Image J | National Institutes of Health | 1.47v |
| GraphPad Prism | GraphPad | 8.0 |
| OriginPro | OriginLab. Inc | 8.5.1 |
| FV10-ASW | Olympus Corp.,Ltd | 04.02.02.09 |
| Pulsemaster software | HEKA | 10.0 |
| Fitmaster analysis software | HEKA | 2X90.3 |
|  |  |  |

**Table S2** Sequences of gene specific siRNA and AAV, and the primers for RT-qPCR.

Table S 2-1 Sequence of gene specific siRNA

| **siRNA oligo** | **Forward** | **Reverse** |
| --- | --- | --- |
| Sortilin | 5’-GAAAGUCAGUGGAAAGUAAdTdT-3’ | 5’-UUACUUUCCACUGACUUUCdTdT-3’ |
|  | 5’-AGUUGGUUGUUAUCCAGAAdTdT-3’ | 5’-UUCUGGAUAACAACCAACUdTdT-3’ |
|  | 5’-GCUGAUAAGGAUACAACAAdTdT-3’ | 5’-UUGUUGUAUCCUUAUCAGCdTdT-3’ |
|  | 5’-GUUCAUGCAUGUAGAUGAAdTdT-3’ | 5’-UUCAUCUACAUGCAUGAACdTdT-3’ |
| TREK1 | 5’-GGCCAUUAAUGUUAUGAAATT-3’ | 5’-UUUCAUAACAUUAAUGGCCTT-3’ |
|  | 5’-GUGGAGGACACAUUUAUUATT-3’ | 5’-UAAUAAAUGUGUCCUCCACTT-3’ |
|  | 5’-GCCGAGUUCAAGGAAACAATT-3’ | 5’-UUGUUUC CUUGAACUGGCTT-3’ |
| TREK2 | 5’-CCUGCUGUCAUCUUUAAAUTT-3’ | 5’-AUUUAAAGAUGACAGCAGGTT-3’ |
|  | 5’-GAGGCCUGCAAACAGUUAUTT-3’ | 5’-AUAACUGUUUGCAGGCCUCTT-3’ |
|  | 5’-CGCACUUGAUGCUGAUAAUTT-3’ | 5’-AUUAUCAGCAUCAAGUGCGTT-3’ |

Table S 2-2 Sequence of gene specific AAV

|  | **GenBank ID** |
| --- | --- |
| Sort1 |  |
|  | 83576（2478bp) |
|  |  |

Table S 2-3 The sequences of primer for RT-qPCR

| **Primer**  **sequence** | **Forward** | **Reverse** |
| --- | --- | --- |
| KCNK2 | 5’-AAggAAgAggTgggAgAgTT-3’ | 5’-CACgCTggAACTTgTCATAgA-3’ |
| KCNK10 | 5’-CTgTTCCTCgACTCTCCATTTC-3’ | 5’-CCACgACCACCACAAAgAT-3’ |
| ACTIN | 5’-AgATCCTgACCgAgCgTggC-3’ | 5’-CCAgggAggAAgAggATgCg-3’ |

**Table S3** Number of samples, normality and equal variance tests, statistical analytical methods and *P* values.

Table S3 Number of samples, normality and equal variance tests, statistical analytical methods and *p* values

| **Figure number** | **Number of animals or samples** | **Normality test / equal variance test** | **Statistic method** | ***p* value** | ***t* value** |
| --- | --- | --- | --- | --- | --- |
| 1C | n =26 cells for control  n =26 cells for prediabetes | failed | *Mann Whitney U* -test | *P=0.001* | *U* =*513.000* |
| 1D | n =18 cells for control  n =14 cells for prediabetes | passed / failed | *Mann Whitney U* -test | *P=0.016* | *U* =*63.000* |
| 1E | n =31 cells for control  n =21 cells for prediabetes | passed / failed | *Mann Whitney U* -test | *P=0.009* | *U* =*185.500* |
| 1F | n =26 cells for control  n =26 cells for prediabetes | failed | *Mann Whitney U* -test | *P=0.018* | *U* =*467.000* |
| 1G | n =26 cells for control  n =21 cells for prediabetes | failed | *Mann Whitney U* -test | *P=0.002* | *U* =*130.000* |
| 1H | n =35 cells for control  n =21 cells for prediabetes |  | Two-way ANOVA RM：  *Mauchly's Test of Sphericity*  𝜒2 = *768.197*  *p* = *0.0001 (*failed)  then, *Greenhouse-Geisser*: *p* =*0.003* (passed)  *F_B_* = *7.202* | control v.s. prediabetes at 100 nA stimulus  *F = 0.704, p =0.405*  control v.s. prediabetes at 200 nA stimulus  *F = 4.785, p = 0.033*  control v.s. prediabetes at 300 nA stimulus  *F = 3.88, p = 0.053*  control v.s. prediabetes at 400 nA stimulus  *F = 5.69, p = 0.021*  control v.s. prediabetes at 500 nA stimulus  *F = 7.74, p = 0.007*  control v.s. prediabetes at 600 nA stimulus  *F = 7.18, p = 0.009*  control v.s. prediabetes at 700 nA stimulus  *F = 6.76, p* = *0.012*  control v.s. prediabetes at 800 nA stimulus  *F =7.66, p* = *0.008*  control v.s. prediabetes at 900 nA stimulus  *F =* *6.58, p* = *0.013*  control v.s. prediabetes at 1000 nA stimulus  *F =* *6.42, p* = *0.014*  control v.s. prediabetes at 1100 nA stimulus  *F =* *9.59, p* = *0.003* |  |
| 1I(right panel) | n =26 cells for control  n =17 cells for prediabetes |  | Two-way ANOVA RM：  *Mauchly's Test of Sphericity*  𝜒2 = *3033.000*  *p* = *0.0001* (failed)  then, *Greenhouse-Geisser: p* =*0.0001*(passed)  *F_B_* = *55.445* | control v.s. prediabetes holding at -80 mV  *F = 2.89, p = 0.097*  control v.s. prediabetes holding at -70 mV  *F =10.28, p = 0.003*  control v.s. prediabetes holding at -60 mV  *F = 8.54, p = 0.006*  control v.s. prediabetes holding at -50 mV  *F = 9.62, p = 0.003*  control v.s. prediabetes holding at -40 mV  *F = 9.00, p = 0.004*  control v.s. prediabetes holding at -30 mV  *F = 8.12, p = 0.007*  control v.s. prediabetes holding at -20 mV  *F = 7.53, p = 0.009*  control v.s. prediabetes holding at -10 mV  *F = 7.16, p = 0.011*  control v.s. prediabetes holding at 0 mV  *F = 6.90, p = 0.012*  control v.s. prediabetes holding at 10 mV  *F = 6.82, p = 0.013*  control v.s. prediabetes holding at 20 mV  *F = 7.69, p = 0.008*  control v.s. prediabetes holding at 30 mV  *F = 8.51, p = 0.006*  control v.s. prediabetes holding at 40 mV  *F = 8.73, p = 0.005*  control v.s. prediabetes holding at 50 mV  *F = 8.67, p = 0.005*  control v.s. prediabetes holding at 60 mV  *F = 8.58, p = 0.006* |  |
| 2D | n =282 cells from 4 rats for control  n =126 cells 4 rats for prediabetes | passed / failed | *Mann Whitney U* -test | *p* =*0.001* | *U* =*1157.500* |
|  | n =115 cells from 4 rats for control  n =96 cells 4 rats for prediabetes | failed | *Mann Whitney U* -test | *p* =*0.0001* | *U* =*493.000* |
|  | n =120 cells from 4 rats for control  n =50 cells 4 rats for prediabetes | failed | *Mann Whitney U* -test | *p* =*0.0001* | *U* = *192.000* |
|  | n =137 cells from 4 rats for control  n =81 cells 4 rats for prediabetes | failed | *Mann Whitney U* -test | *p =0.0001* | *U* = *2384.500* |
| 2F | n =4 rats for control  n =4 rats for prediabetes | passed/passed | *t-* test | *p* =*0.002* | *t* =*5.196* |
|  | n =5 rats for control  n =5 rats for prediabetes | passed/passed | *t-* test | *p* =*0.003* | *t* =*4.197* |
| 3D | n =257 cells from 4 rats for control  n =139 cells 4 rats for prediabetes | failed | *Mann Whitney U* -test | *p =0.0001* | *U* =*719.500* |
|  | n =127 cells from 4 rats for control  n =98 cells 4 rats for prediabetes | passed / failed | *Mann Whitney U* -test | *p =0.0001* | *U* =*62.500* |
|  | n =67 cells from 4 rats for control  n =54 cells 4 rats for prediabetes | failed | *Mann Whitney U* -test | *p =0.005* | *U* =*1270.000* |
|  | n =105 cells from 4 rats for control  n =91 cells 4 rats for prediabetes | failed / passed | *Mann Whitney U* -test | *p =0.0001* | *U* =*426.500* |
| 3F | n =4 rats for control  n =4 rats for prediabetes | passed/passed | *t-* test | *p* =*0.006* | *t* =*4.188* |
|  | n =5 rats for control  n =5 rats for prediabetes | passed/passed | *t-* test | *p* =*0.005* | *t* =*3.852* |
| 4B | n =4 rats for siRNA-sham  n =4 rats for siRNA-TREK1 | passed/passed | *t-* test | *p* =*0.021* | *t* =*3.102* |
|  | n =4 rats for siRNA-sham  n =4 rats for siRNA-TREK2 | passed/passed | *t-* test | *p* =*0.001* | *t* =*6.073* |
| 4C  (left panel) | n =8 rats for siRNA-sham  n =5 rats for siRNA-TREK1 | failed | *Mann Whitney U* -test | *p =0.045* | *U* =*6.500* |
|  | n =8 rats for siRNA-sham  n =6 rats for siRNA-TREK1 | failed / passed | *Mann Whitney U* -test | *p =0.0001* | *U* < *0.0001* |
|  | n =8 rats for siRNA-sham  n =6 rats for siRNA-TREK1 | passed/ failed | *Mann Whitney U* -test | *p =0.001* | *U* < *0.0001* |
| 4C  (right panel) | n =8 rats for siRNA-sham  n =7 rats for siRNA-TREK1 | passed/passed | *t-* test | *p =0.021* | *t =2.629* |
|  | n =8 rats for siRNA-sham  n =7 rats for siRNA-TREK1 | passed/passed | *t-* test | *p =0.0001* | *t =5.040* |
| 4D  (left panel) | n =7 rats for siRNA-sham  n =10 rats for siRNA-TREK2 | passed/passed | *t-* test | *p =0.158* | *t =1.485* |
|  | n =7 rats for siRNA-sham  n =10 rats for siRNA-TREK2 | passed/passed | *t-* test | *p =0.0001* | *t =4.805* |
|  | n =7 rats for siRNA-sham  n =7 rats for siRNA-TREK2 | passed/passed | *t-* test | *p =0.0001* | *t =9.427* |
| 4D  (right panel) | n =8 rats for siRNA-sham  n =8 rats for siRNA-TREK2 | passed/passed | *t-* test | *p =0.001* | *t =3.951* |
|  | n =8 rats for siRNA-sham  n =7 rats for siRNA-TREK2 | passed/passed | *t-* test | *p =0.0001* | *t =5.144* |
| 5D | n =215 cells from 4 rats for control  n =115 cells 4 rats for prediabetes | failed | *Mann Whitney U* -test | *p =0.0001* | *U* =*719.500* |
|  | n =32 cells from 4 rats for control  n =142 cells 4 rats for prediabetes | failed | *Mann Whitney U* -test | *p =0.0001* | *U* =*4295.500* |
|  | n =135 cells from 4 rats for control  n =62 cells 4 rats for prediabetes | failed | *Mann Whitney U* -test | *p =0.0001* | *U* =*6091.500* |
|  | n =119 cells from 4 rats for control  n =209 cells 4 rats for prediabetes | failed | *Mann Whitney U* -test | *p =0.0001* | *U* =*16204.000* |
| 5F | n =4 rats for control  n =4 rats for prediabetes | passed/passed | *t-* test | *p =0.002* | *t =-5.049* |
|  | n =5 rats for control  n =5 rats for prediabetes | passed/passed | *t-* test | *p =0.0001* | *t =-5.796* |
| 6A(right panel) | n =22 cells from 4 rats for control  n =11 cells 4 rats for prediabetes | passed/passed | *t-* test | *p =0.551* | *t =-0.603* |
|  | n =16 cells from 4 rats for control  n =18 cells 4 rats for prediabetes | failed | *Mann Whitney U* -test | *p =0.084* | *U* =*60.000* |
| 6C | n =6 rats for vehicle  n =7 rats for Spadin 20μᴍ injection |  | Two-way ANOVA RM：  *Mauchly's Test of Sphericity*  𝜒2 = *17.845*  *p = 0.04* (failed)  then, *Greenhouse-Geisser: p* =0.01 (passed)  *F_A,B_* = *4.793* | vehicle *v.s.* spadin 20 μM injection at 0 min  *F = 0.013, p = 0.91*  vehicle *v.s.* spadin 20 μM injection at 30 min  *F = 15.758, p = 0.002*  vehicle *v.s.* spadin 20 μM injection at 1 h  *F = 14.858, p = 0.003*  vehicle *v.s.* spadin 20 μM injection at 2 h  *F = 13.910, p = 0.003*  vehicle *v.s.* spadin 20 μM injection at 2 h  *F = 16.360, p = 0.002* |  |
| 6D | n =12 rats for vehicle  n =7 rats for Spadin 10μᴍ injection  n =7 rats for Spadin 20μᴍ injection | failed | *Kruskal-Wallis* *with Bonferroni post hoc correction* | vehicle *v.s.* spadin 10μM injection  *p* = 0.003  vehicle *v.s.* spadin 20μM injection  *p* = 0.0001  spadin 10μM injection *v.s.* spadin 20μM injection  *p* = 1.000 | *H* =19.472 |
| 6E | n =12 rats for vehicle  n =7 rats for Spadin 20μᴍ injection | passed/passed | *t-* test | *p =0.001* | *t =4.922* |
| 6G | n =4 rats for vehicle  n =4 rats for Spadin 20μᴍ injection | passed/passed | *t-* test | *p =0.006* | *t =4.097* |
|  | n =4 rats for vehicle  n =4 rats for Spadin 20μᴍ injection | passed/passed | *t-* test | *p =0.001* | *t =5.619* |
| 7B | n = 5 rats for AAV-sham  n = 5 rats for AAV-Sort1 | passed/passed | *t-* test | *p =0.042* | *t =-2.420* |
|  | n = 5 rats for AAV-sham  n = 5 rats for AAV-Sort1 | passed/passed | *t-* test | *p =0.001* | *t =5.525* |
|  | n = 5 rats for AAV-sham  n = 5 rats for AAV-Sort1 | passed/passed | *t-* test | *p =0.001* | *t =5.029* |
| 7D | n = 55 cells from 4 rats for  AAV-sham  n = 128 cells from 4 rats for  AAV-Sort1 | failed | *Mann Whitney U* -test | *p =0.0001* | *U* =*7039.000* |
|  | n = 150 cells from 4 rats for  siRNA-sham  n = 47 cells from 4 rats for  siRNA-Sort1 | failed | *Mann Whitney U* -test | *p =0.0001* | *U* =*3.000* |
|  | n = 96 cells from 4 rats for  siRNA-sham  n = 107 cells from 4 rats for  siRNA-Sort1 | failed | *Mann Whitney U* -test | *p =0.0001* | *U* =*439.500* |
| 7E | n = 6 rats for control  n = 7 rats for AAV-sham | failed | *Mann Whitney U* -test | *p =0.836* | *U* =*19.000* |
|  | n = 6 rats for AAV-sham  n = 6 rats for AAV-Sort1 | failed | *Mann Whitney U* -test | *p =0.002* | *U <*0.0001 |
| 7F | n = 6 rats for control  n = 7 rats for AAV-sham | failed | *Mann Whitney U* -test | *p =0.445* | *U* =*15.000* |
|  | n = 6 rats for AAV-sham  n = 6 rats for AAV-Sort1 | failed | *Mann Whitney U* -test | *p =0.132* | *U* =*8.000* |
| 8B | n = 167 cells from 4 rats for  siRNA-sham  n = 135 cells from 4 rats for  siRNA-Sort1 | failed | *Mann Whitney U* -test | *p =0.0001* | *U* =*182.000* |
|  | n = 82 cells from 4 rats for  siRNA-sham  n = 125 cells from 4 rats for  siRNA-Sort1 | failed | *Mann Whitney U* -test | *p =0.0001* | *U* =*9100.000* |
|  | n = 81 cells from 4 rats for  siRNA-sham  n = 129 cells from 4 rats for  siRNA-Sort1 | failed | *Mann Whitney U* -test | *p =0.0001* | *U* =*8757.500* |
| 8D | n = 5 rats for siRNA-sham  n = 5 rats for siRNA-Sort1 | passed/passed | *t-* test | *p =0.008* | *t =3.510* |
|  | n = 5 rats for siRNA-sham  n = 5 rats for siRNA-Sort1 | passed/passed | *t-* test | *p =0.004* | *t =-4.612* |
|  | n = 5 rats for siRNA-sham  n = 5 rats for siRNA-Sort1 | passed/passed | *t-* test | *p =0.009* | *t =-3.391* |
| 8E | n = 8 rats for siRNA-sham  n = 12 rats for siRNA-Sort1 | failed | *Mann Whitney U* -test | *p =0.025* | *U* =*76.500* |
|  | n = 8 rats for siRNA-sham  n = 10 rats for siRNA-Sort1 | failed | *Mann Whitney U* -test | *p =0.0001* | *U* =*79.500* |
|  | n = 8 rats for siRNA-sham  n = 9 rats for siRNA-Sort1 | failed | *Mann Whitney U* -test | *p =0.0001* | *U* =*72.000* |
| 8F | n = 6 rats for siRNA-sham  n = 8 rats for siRNA-Sort1 | passed/passed | *t-* test | *p =0.013* | *t =-2.923* |
|  | n = 6 rats for siRNA-sham  n = 9 rats for siRNA-Sort1 | passed/passed | *t-* test | *p =0.035* | *t =-2.355* |
| 9A  （middle pannel） | n = 15 cells for control  n = 7 cells for prediabetes |  | Two-way ANOVA RM*：*  *Mauchly's Test of Sphericity*  *𝜒2 = 1030.842*  *p = 0.0001 (failed)*  then*, Greenhouse-Geisser: p =0.006 (passed)*  *F_B_ = 8.721* | control *v.s.* prediabetes holding at -80 mV  *F = 3.634, p = 0.05*  control *v.s.* prediabetes holding at -70 mV  *F =4.450, p = 0.025*  control *v.s.* prediabetes holding at -60 mV  *F = 5.590, p = 0.012*  control *v.s.* prediabetes holding at -50 mV  *F = 5.548, p = 0.012*  control *v.s.* prediabetes holding at -40 mV  *F = 6.147 p = 0.008*  control *v.s.* prediabetes holding at -30 mV  *F = 7.043, p = 0.005*  control *v.s.* prediabetes holding at -20 mV  *F = 7.978, p = 0.003*  control *v.s.* prediabetes holding at -10 mV  *F = 10.076, p = 0.001*  control *v.s.* prediabetes holding at 0 mV  *F = 9.923, p = 0.001*  control *v.s.* prediabetes holding at 10 mV  *F = 9.379, p = 0.001*  control *v.s.* prediabetes holding at 20 mV  *F = 11.879, p = 0.0001*  control *v.s.* prediabetes holding at 30 mV  *F = 10.891, p = 0.001*  control *v.s.* prediabetes holding at 40 mV  *F = 10.216, p = 0.001*  control *v.s.* prediabetes holding at 50 mV  *F = 9.358, p = 0.001*  control *v.s.* prediabetes holding at 60 mV  *F = 8.532, p = 0.002* |  |
| 9D | n = 6 cells for prediabetes  n = 6 cells for +BL1249 | passed/passed | *Paired t-test* | *p =0.146* | *t =1.720* |
| 9E | n = 6 cells for prediabetes  n = 6 cells for +BL1249 | passed/passed | *Paired t-test* | *p =0.0001* | *t =-10.448* |
| 9F | n = 6 cells for prediabetes  n = 6 cells for +BL1249 | passed/passed | *Paired t-test* | *p =0.016* | *t =-3.567* |
| 9G | n = 6 cells for prediabetes  n = 6 cells for +BL1249 | failed | *Paired Samples Wilcoxon Signed Rank Test* | *p =0.028* | *Z =-2.201* |
| 9H | n = 6 cells for prediabetes  n = 6 cells for +BL1249 | passed/passed | *Paired t-test* | *p =0.072* | *t =2.279* |
| 9I | n = 6 cells for prediabetes  n = 6 cells for +BL1249 | failed | *Paired Samples Wilcoxon Signed Rank Test* | *p =0.031* | *Z =-2.041* |
| 9J | n = 9 cells for prediabetes  n = 9 cells for +BL1249 |  | Two-way ANOVA RM：  *Mauchly's Test of Sphericity*  𝜒2 = *335.331*  *p* = *0.0001* (failed)  then, *Greenhouse-Geisser:* *p* = *0.03* (passed)  *F_A,B_* = 8.346 | prediabetes *v.s.* +BL1249 at 200 pA stimulus  *F = 2.222, p = 0.155*  prediabetes *v.s.* +BL1249 at 400 pA stimulus  *F = 2.086, p = 0.168*  prediabetes *v.s.* +BL1249 at 600 pA stimulus  *F =3.913, p = 0.065*  prediabetes *v.s.* +BL1249 at 800 pA stimulus  *F = 4.804, p = 0.044*  prediabetes *v.s.* +BL1249 at 1000 pA stimulus  *F = 6.879, p = 0.018*  prediabetes *v.s.* +BL1249 at 1200 pA stimulus  *F = 8.493, p = 0.010*  prediabetes *v.s.* +BL1249 at 1400 pA stimulus  *F = 10.432, p = 0.005*  prediabetes *v.s.* +BL1249 at 1600 pA stimulus  *F = 11.808, p = 0.003* |  |
| S1A | n = 10 rats for control  n = 10 rats for high energy diet | passed/passed | *t-* test | control v.s. high energy diet at 25 d  *t =1.213, p =0.241*  control v.s. high energy diet at 50 d  *t =-1.060, p =0.303*  control v.s. high energy diet at 65 d  *t =0.350, p =0.730* |  |
| S1B | n = 10 rats for control  n = 10 rats for high energy diet | passed/passed | *t-* test | control v.s. high energy diet at 25 d  *t =-0.138, p =0.892*  control v.s. high energy diet at 65 d  *t =0.360, p =0.723* |  |
| S1C | n = 10 rats for control  n = 10 rats for high energy diet  (7 rats for high energy diet at 80 d) | failed | *Mann Whitney U* -test | control v.s. high energy diet at 25 d  *U =31.000, p =0.165*  control v.s. high energy diet at 65 d  *U =92.000, p =0.001*  control v.s. high energy diet at 80 d  *U =70.000, p =0.0001* |  |
| S1D | n = 10 rats for control  n = 10 rats for high energy diet  (7 rats for high energy diet at 80 d) | passed/passed | *t-* test | control v.s. high energy diet at 25 d  *t =1.094, p =0.289* |  |
|  |  | failed | *Mann Whitney U* -test | control v.s. high energy diet at 65 d  *U =80.000, p =0.023*  control v.s. high energy diet at 80 d  *U =69.000, p =0.0001* |  |
| S2A | n = 20 rats for control  n = 20 rats for high energy diet (17 rats for high energy diet at 80 d) | failed | *Mann Whitney U* -test | control v.s. high energy diet at 25 d  *U =170.500, p =0.429*  control v.s. high energy diet at 50 d  *U =69.000, p =0.0001*  control v.s. high energy diet at 65 d  *U =62.500, p =0.0001*  control v.s. high energy diet at 80d  *U =1.500, p =0.0001* |  |
| S2B | n = 20 rats for control  n = 20 rats for high energy diet (17 rats for high energy diet at 80 d) | failed | *Mann Whitney U* -test | control v.s. high energy diet at 25 d  *U =168.000, p =0.398*  control v.s. high energy diet at 50 d  *U =89.500, p =0.002*  control v.s. high energy diet at 65 d  *U =41.500, p =0.0001*  control v.s. high energy diet at 80d  *U =13.000, p =0.0001* |  |
| S2C | n = 20 rats for control  n =20 rats for high energy diet | failed | *Mann Whitney U* -test | *p =0.001* | *U =83.000* |
| S2D | n = 20 rats for control  n = 20 rats for high energy diet | failed | *Mann Whitney U* -test | *p =0.005* | *U =97.500* |
| S3 | KCNN2:  n = 4 rats for vehicle  n = 4 rats for Spadin 20μᴍ injection | passed/passed | *t-test* | *p =0.402* | *t =-0.902* |
|  | KCNN10:  n = 4 rats for vehicle  n = 4 rats for Spadin 20μᴍ injection | passed/passed | *t-test* | *p =0.427* | *t =-0.852* |
| S4A | n = 6 rats for prediabetes  n = 6 rats for +BL1249 |  | Two-way ANOVA RM：  *Mauchly's Test of Sphericity*  𝜒2 = *6.380*  *p* = *0.274* (passed) | prediabetes v.s. +BL1249 before injection  *F = 0.172, p = 0.687*  prediabetes v.s. +BL1249 at 90 min after injection  *F = 9.666, p = 0.011*  prediabetes v.s. +BL1249 at 240 min after injection  *F = 6.692, p = 0.027*  prediabetes v.s. +BL1249 at 360 min after injection  *F = 11.201, p = 0.007* |  |
| S4B | n = 6 rats for prediabetes  n = 6 rats for +BL1249 | passed/passed | *t-test* | *p =0.011* | *t =-3.120* |
| S5B  (left pannel) | n =3 rats for siRNA sham  n =3 rats for siRNA TREK2 | passed/passed | *t-test* | *p =0.861* | *t =-0.186* |
| S5B  (right pannel) | n =3 rats for siRNA sham  n =3 rats for siRNA TREK1 | failed | *Mann Whitney U* -test | *p =0.700* | *U =3.000* |
|  |  |  |  |  |  |
|  |  |  |  |  |  |
